# Cherry picking by pseudomonads: After a century of research on canker, genomics provides insights into the evolution of pathogenicity towards stone fruits

**DOI:** 10.1111/ppa.13189

**Published:** 2020-05-06

**Authors:** Michelle T. Hulin, Robert W. Jackson, Richard J. Harrison, John W. Mansfield

**Affiliations:** ^1^ NIAB EMR East Malling UK; ^2^ Birmingham Institute of Forest Research (BIFoR), University of Birmingham Birmingham UK; ^3^ School of Biosciences, University of Birmingham Birmingham UK; ^4^ NIAB Cambridge UK; ^5^ Faculty of Natural Sciences Imperial College London London UK

**Keywords:** evolution, pathogenicity, *Prunus*, *Pseudomonas*

## Abstract

Bacterial canker disease is a major limiting factor in the growing of cherry and other *Prunus* species worldwide. At least five distinct clades within the bacterial species complex *Pseudomonas syringae* are known to be causal agents of the disease. The different pathogens commonly coexist in the field. Reducing canker is a challenging prospect as the efficacy of chemical controls and host resistance may vary against each of the diverse clades involved. Genomic analysis has revealed that the pathogens use a variable repertoire of virulence factors to cause the disease. Significantly, strains of *P*. *syringae* pv. *syringae* possess more genes for toxin biosynthesis and fewer encoding type III effector proteins. There is also a shared pool of key effector genes present on mobile elements such as plasmids and prophages that may have roles in virulence. By contrast, there is evidence that absence or truncation of certain effector genes, such as *hopAB*, is characteristic of cherry pathogens. Here we highlight how recent research, underpinned by the earlier epidemiological studies, is allowing significant progress in our understanding of the canker pathogens. This fundamental knowledge, combined with emerging insights into host genetics, provides the groundwork for development of precise control measures and informed approaches to breed for disease resistance.

## Graphical abstract

A review of *Pseudomonas* cherry canker linking genomics to control.

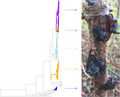

## 1 BACKGROUND

*Pseudomonas syringae* is a bacterial species complex associated with plants and aquatic environments that has been reported to cause disease on over 180 plant species (Berge *et al.*, 2014). The pathogen is important globally as it infects most major crops. Host‐adapted strains are responsible for damaging disease epidemics when invading new territories, for example the outbreak of horse chestnut bleeding canker in northern Europe (Green *et al.*, 2010; Steele *et al.*, 2010) and kiwifruit canker in New Zealand (McCann *et al.*, 2017). *P*.* syringae* strains were traditionally classified based on host of isolation into groups of pathogenic varieties (pathovars) that generally infect one or a few related plant species (Sarkar *et al.*, 2006). Classification is now supported by genotypic data, leading to the proposal for distinct genomospecies, phylogroups or phylogenomic species based on DNA–DNA hybridization, multilocus sequence analysis (MLSA) or more recently, whole genome sequence data comparisons (Gomila *et al.*, 2017). Nineteen phylogenomic species have been defined within the complex, which generally correspond to the described phylogroups (Gomila *et al.*, 2017). Strains of *P*.* syringae *pv.* syringae *(Pss), *P*.* syringae *pv.* morsprunorum* (Psm) race 1 (R1) and Psm race 2 (R2) are recognized as the principle cause of bacterial canker on *Prunus* species (Bultreys and Kałużna, 2010).

Since it was first reported in the early 1900s (Brzezinski, 1902), bacterial canker has contributed to major production losses worldwide (Kennelly *et al.*, 2007). It is a serious problem for young plantations and nurseries, and cherry tree losses of up to 75% have been reported in Oregon due to the disease (Spotts *et al.*, 2010). Spraying with copper‐based biocides is the major control method; however, this is being phased out in Europe and strains with copper resistance are frequently reported (Sundin and Bender, 1993; Ghorbani and Wilcockson, 2007). No fully resistant host varieties have been identified. Resistance breeding is also complicated by the remarkable diversity of bacteria causing the disease (Spotts *et al.*, 2010; Farhadfar *et al.*, 2016).

**FIGURE 1:**
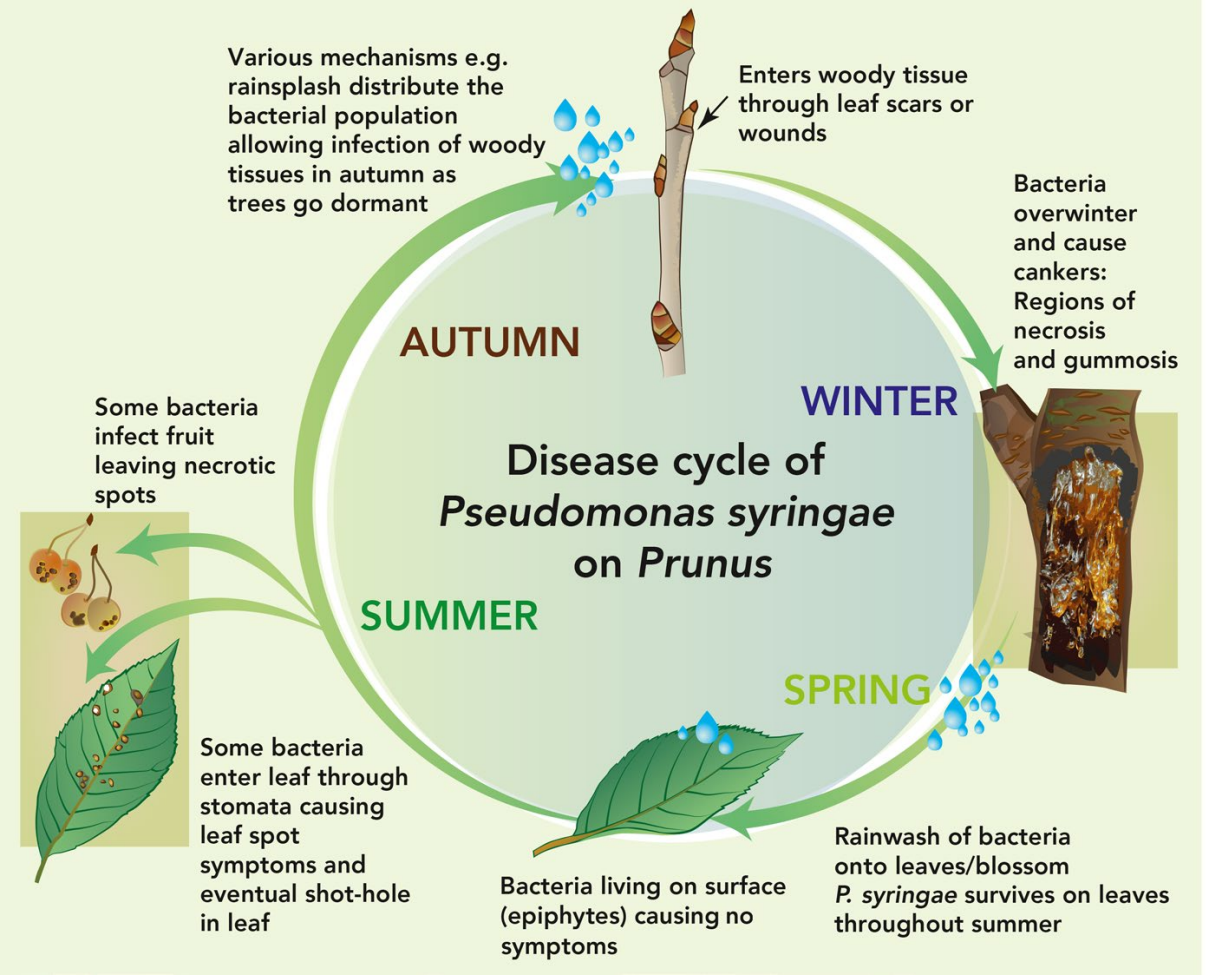
Overview of the canker disease cycle on *Prunus* based primarily on Crosse (1966), and Crosse and Garrett (1966)

The genus *Prunus* contains economically important species such as cherry, plum, almond, apricot, and peach, all of which are vulnerable to *Pseudomonas *diseases. Bacteria infect all aerial plant organs throughout the season, causing fruit spot, shoot necrosis, and blossom blight, as well as causing leaf spot symptoms where the tissue can drop out to leave shot‐holes (Crosse, 1966). The disease becomes most serious when bacteria infect woody tissues, causing black necrotic cankers and dieback that may lead to tree death. During the growing period, a diverse population of epiphytic bacteria survive on the leaf surface. This population, that may not induce symptoms provides the inoculum for leaf scar and wound infections in the autumn (Crosse, 1959). The pathogens exist in several different niches on and within the host at various stages of the disease cycle (Figure 1).

This review highlights how recent genome‐based research has brought this and other woody plant‐infecting bacterial pathogens into focus, and could stimulate development of novel diagnostics, precise control methods, and accelerated resistance breeding in woody perennials.

## 2 IDENTIFYING THE CAUSAL AGENTS OF BACTERIAL CANKER

Research on the disease in the UK began at East Malling in the 1920s, with the pioneering work of Dr Harry Wormald, who noticed a dieback disease in plum was caused by at least two species of bacteria that entered through wounds (Wormald, 1930). He named one of the species *Pseudomonas morsprunorum*, and the other *Pseudomonas prunicola* (Wormald, 1932). It was later determined that *P*. *prunicola* is Pss and that *P*. *morsprunorum* is a pathovar within *P*. *syringae* (Crosse, 1966). Following this original work, strains of Psm were divided into two races (Psm R1 and Psm R2) based on biochemical markers and differential pathogenicity on host genotypes (Freigoun and Crosse, 1975). Genetic data has subsequently shown that the two races are distantly related and should not be considered as races of a single pathovar (Bultreys and Kałużna, 2010). Other pseudomonads closely related to Pss, Psm R1, or Psm R2 have recently been associated with cankers of cherry, namely *P*. *cerasi* and *P*. *syringae* pv. *avii* (Ménard *et al.*, 2003; Kałużna *et al.*, 2016b). *P*. *viridiflava* strains have also been isolated from cankerous shoot tissues of various *Prunus* species (Harzallah *et al.*, 2004; Parisi *et al.*, 2019). Cherry and relatives in the *Prunus* subgenus *Cerasus* are additionally susceptible to bacterial gall disease caused by *P*. *syringae* pv. *cerasicola* (Kamiunten *et al.*, 2000). Aside from cherry, cankers are also associated with the clades *P*. *syringae* pv. *persicae* on peach and *P*. *amygdali* on almond (Psallidas and Panagopoulos, 1975; Young, 2010).

**FIGURE 2:**
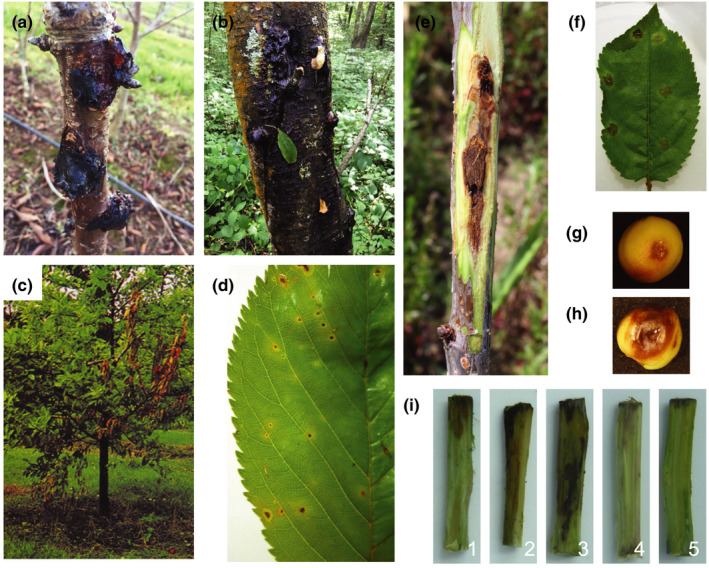
Examples of natural and artificial infection of *Pseudomonas syringae* disease symptoms on *Prunus*. (a) Natural infection of cherry cv. Van in an orchard in Kent, UK. Picture taken by M. Hulin in 2017. (b) Wild cherry infection in a forest in Kent, UK. Picture taken by M. Hulin in 2019. (c) Dieback of plum, picture taken by C. M. E. Garrett in 1980. (d) Leaf spots due to natural infection on cherry cv. Napoleon from an orchard in Kent, UK. Picture taken by M. Hulin in 2014. (e) Artificial inoculation of cherry cv. Van with *Pseudomonas syringae* pv. *syringae* (Pss) 9293, picture taken 6 months after inoculation after stripping back the bark. (f) Leaf infiltrated with *P*. *syringae* pv. *morsprunorum* (Psm) R1‐5244 showing symptoms after 7 days. (g) Immature cherry fruit inoculated with Psm R1‐5244. (h) Immature cherry fruit inoculated with Pss 9097. (i) Cut shoot inoculation of Pss 9097 on cherry cv. Napoleon (1–4) and a negative control (10 mM MgCl_2_) (5). Images taken after 6 weeks with bark stripped back. Shoot images are from Hulin *et al. *(2018b)

The epidemiology of this complex disease has previously been reviewed in detail (Crosse, 1966; Kennelly *et al.*, 2007; Bultreys and Kałużna, 2010; Konavko *et al.*, 2014). Unravelling the pathogen's disease cycle (Figure 1) has greatly informed control strategies. Figure 2 shows symptoms of natural and artificial infection. *P*. *syringae* clades that cause bacterial canker in *Prunus* may differ in lifestyle and aggressiveness on different host tissues, reflecting the multiple niches that perennial crops provide. For example, leaf scars may be the dominant route of infection of cherry by Psm R1 but not by Pss, which preferentially invades through wounds (Cameron, 1962; Crosse and Garrett, 1966). Additional information also suggested that Pss cannot survive in cankers as long as Psm R1 (Crosse and Garrett, 1966); however, it belongs to a phylogroup with strains characterized as better epiphytes (Feil *et al.*, 2005; Helmann *et al.*, 2019), so may be more suited for colonization of the plant surface. Environmental factors such as frost occurrence can also significantly increase the incidence of infection (Kennelly *et al.*, 2007), presumably due to wound creation by frost damage. Inoculation of immature cherry fruits has also revealed differences in induced symptoms, with Pss strains causing large black necrotic lesions whilst Psm races 1 and 2 cause small water‐soaked lesions (Bultreys and Kałużna, 2010). Within each pathovar, strains may also exhibit differences in aggressiveness towards a particular host species within *Prunus* (Gilbert *et al.*, 2009; Hulin *et al.*, 2018b), indicating there is significant variation even between closely related strains equivalent to differentiation into physiological races.

## 3 PHYLOGENETICS REVEALS CONVERGENT EVOLUTION

The different clades of *P*. *syringae* and other *Pseudomonas* species associated with various *Prunus* diseases, their geographical distribution, associated symptoms and known host range are summarized in Table 1. Significantly, sampling studies have indicated that the different pathogens commonly coexist within orchards, nurseries, and forests (Vicente *et al.*, 2004; Gilbert *et al.*, 2008; Kałużna *et al.*, 2010).

**TABLE 1:** Host range and geographical distribution of major *Pseudomonas* species that cause disease on *Prunus*

Species	*P. syringae* pathovar	Proposed phylogenomic species^c^	Phylogroup	Hosts	Associated symptoms	Known distribution	References
*P. syringae*	*morsprunorum* R1	*P. amygdali*	3	*P. avium* ^a^ *, P. cerasus* ^a^ *, P. domestica* ^a^ *, P. armeniaca* ^a^ *, P. persica, P. amydalus* ^b^	Canker, dieback, necrosis on leaves, fruit, blossom	Europe, C. America, S. Africa, N. America, Australasia	Wormald (1932, 1937, 1942); Crosse (1953); Latorre and Jones (1979); Sundin *et al. *(1988); Gilbert *et al. *(2009); Giovanardi *et al. *(2018); Hulin *et al. *(2018b); Ahmed *et al. *(2018); Parisi *et al. *(2019)
*P. syringae*	*morsprunorum* R2	*P. avellanae*	1b	*P. avium* ^a^ *, P. cerasus* ^a^ *, P. domestica* ^a^ *, P. armeniaca*	Canker, dieback, necrosis on leaves, fruit, blossom	Europe, S. Africa	Freigoun and Crosse (1975); Sulikowska and Sobiczewski (2008); Gilbert *et al. *(2009); Giovanardi *et al. *(2018); Hulin *et al. *(2018b)
*P. syringae*	*syringae*	*P. syringae*	2b, 2d	*P. avium* ^a^ *, P. cerasus* ^a^ *, P. domestica* ^a^ *, P. armeniaca* ^a^ *, P. persica* ^a^ *, P. amydalus*	Canker, dieback, necrosis on leaves, fruit, blossom	Europe, C. America, S. America, S. Africa, N. America, C. Asia, Australasia	Wilson (1939); Sundin *et al. *(1988); Gilbert *et al. *(2009); Kałużna *et al. *(2010); Rezaei and Taghavi (2014); Giovanardi *et al. *(2018); Hulin *et al. *(2018b); Bophela *et al. *(2020); Parisi *et al. *(2019)
*P. syringae*	*avii*	*P. tomato*	1a	*P. avium* ^a^	Canker	Europe	Ménard *et al. *(2003)
*P. cerasi*		*P. cerasi*	2a	*P. avium, P. cerasus*	Canker and necrosis of shoots, necrosis on leaves, fruit, blossom	Europe	Kałużna *et al. *(2016b)
*P. amygdali*		*P. amygdali*	3	*P. amygdalus* ^a^	Hyperplastic canker	Europe, C. Asia	Psallidas and Panagopoulos (1975)
*P. syringae*	*persicae*	*P. tomato*	1a	*P. persica* ^a^ *, P. salicina* ^ad^ *, P. domestica* ^ad^ *,P. cerasifera* ^a^	Canker and decline, necrosis of leaves, fruit	Europe, Australasia	Young (1987, 1995)
*P. syringae*	*cerasicola*	*P. amygdali*	3	*Prunus* × *yedoensis* ^a^ *, P. avium* ^a^ *, P. armeniaca* ^a^ *, P. cerasoides* ^a^ *, P. jamasakura* ^a^ *, P. leveilleana* ^a^ *, P. sargentii* ^a^	Bacterial gall	E. Asia	Kamiunten *et al. *(2000)
*P. viridiflava*		*P. viridiflava*	7	*P. avium, P. cerasus, P. domestica* ^a^ *, P. armeniaca* ^a^ *, P. persica* ^a^	Canker, shoot necrosis, tree apoplexy during growing season	Europe, Africa	Scortichini and Morone (1997); Harzallah *et al. *(2004); Sulikowska and Sobiczewski (2008); Bophela *et al. *(2020); Parisi *et al. *(2019)

^a^ Confirmed pathogenicity on host through controlled pathogenicity tests.

^b^ Unsure if R1 as only based on morphological data.

^c^ Phylogenomic species as defined in Gomila *et al. *(2017).

^d^ Weak pathogen.

**FIGURE 3:**
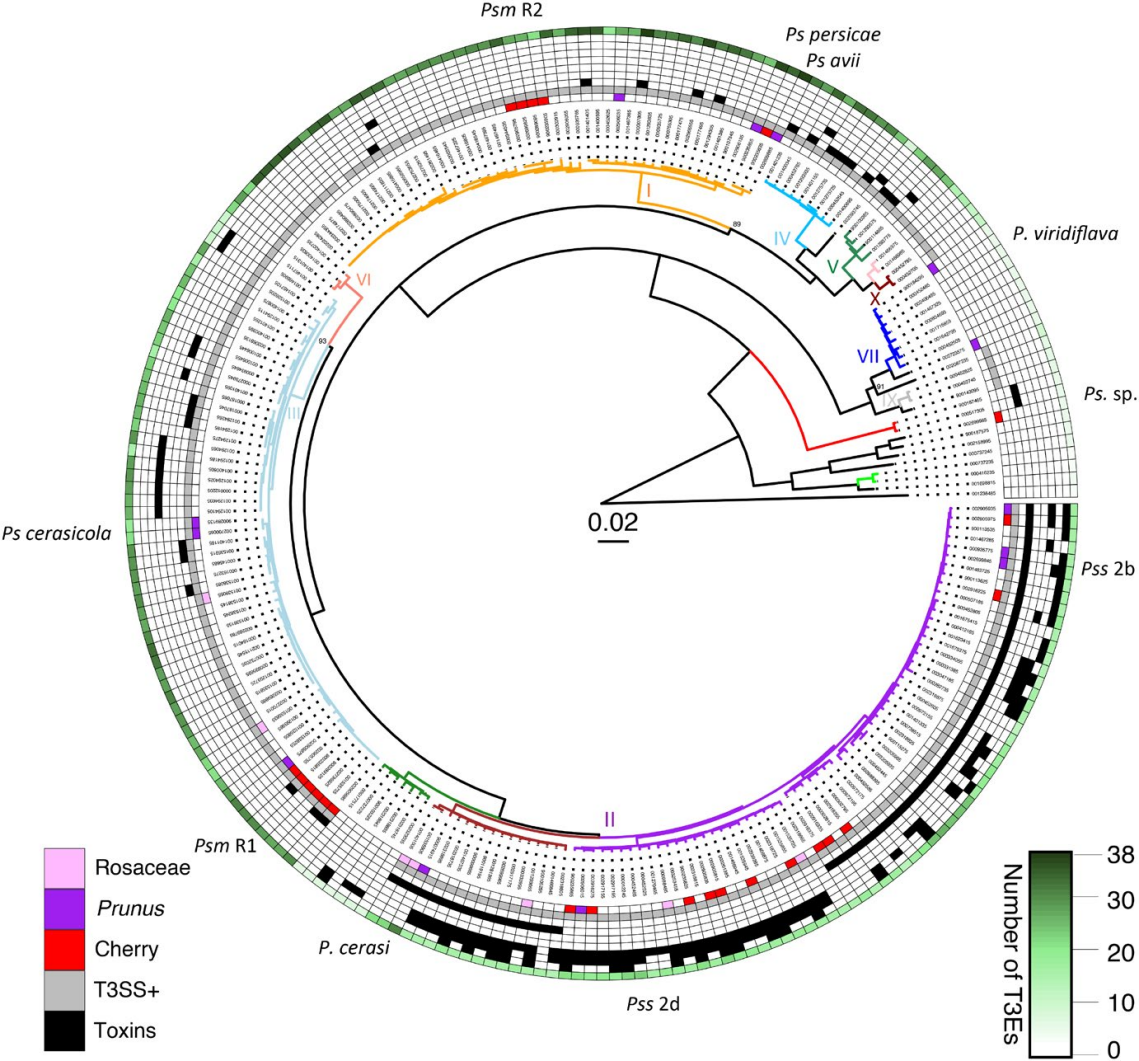
Core genome phylogenetic tree of *Pseudomonas syringae* species complex highlighting strains isolated from *Prunus*. The tree was generated using IQ‐TREE (Nguyen *et al.*, 2015). Phylogenomic clades based on whole genome average nucleotide identity (ANI) of ≥95% are coloured and phylogroups numbered. Circles going outwards: (1) strains isolated from cherry, other *Prunus* and Rosaceae are coloured according to the scale; (2) presence of the canonical type III secretion system (T3SS) in grey (see Text S1 and Table S3 for scoring criteria); (3–9) toxin biosynthesis gene presence in black (order: coronatine, mangotoxin, tabtoxin, phaseolotoxin, syringolin A, syringomycin, syringopeptin). Outer circle (10) a heatmap of number of predicted type III effectors (T3Es, based on known characterized families) scaled from white to dark green. Bootstrap support values under 99% are shown for inner branches. The scale shows substitutions per site

Core genome phylogenetic analysis (Figure 3) reveals the remarkable diversity of bacteria causing canker symptoms in cherry and other *Prunus* spp., with strains falling into four phylogroups (Gilbert *et al.*, 2008; Bultreys and Kałużna, 2010; Hulin *et al.*, 2018a; Parisi *et al.*, 2019; Ruinelli *et al.*, 2019). Psm R1 and R2 have evolved within woody host‐infecting clades of phylogroups 3 and 1, respectively (Nowell *et al.*, 2016). *P*. *syringae* pv. *cerasicola*, which is the causal agent of bacterial gall, is also in phylogroup 3 with Psm R1, but is within a different clade, more closely related to *P*. *syringae* pv. *ulmi*, a pathogen of elm. *P*. *syringae* pv. *avii* is within the same phylogroup as Psm R2, but is more closely related to strains belonging to *P*. *syringae* pv. *persicae*. *P*. *cerasi* is within phylogroup 2a (although it is proposed as a new phylogenomic species; Gomila *et al.*, 2017) and is closely related to the apple blister spot pathogen *P*. *syringae* pv. *papulans* (Hanvantari, 1977). By contrast, strains classified as Pss are highly diverse, originating in different subphylogroups within phylogroup 2 (2b and 2d) and interspersed with Pss pathogens of various plant species and also strains present in the wider environment such as water sources (Ahmadi *et al.*, 2017). *P*. *viridiflava* falls within phylogroup 7, with strains isolated from various plants and the environment (Bartoli *et al.*, 2014).

The designation of strains as pathovar *syringae* (i.e., Pss) within phylogroup 2 provides no information about their host range or symptoms produced, and ignores genetic diversity. In terms of host specificity, closely related strains of Pss have been isolated from different *Prunus* species, such as apricot, cherry, and plum and many can cross‐infect (Gilbert *et al.*, 2008; Rezaei and Taghavi, 2014; Hulin *et al.*, 2018b). Members of the other *Prunus*‐infecting clades may be more host‐restricted. In particular, Psm R1 strains can be differentiated into at least two host‐specific lineages that show differential virulence on *Prunus* species (Crosse, 1953; Crosse and Garrett, 1970; Hulin *et al.*, 2018a). Phylogenomics revealed two closely related sister groups within this clade that are >99.7% identical at the genome level (M. T. Hulin, unpublished observation), using the program PYANI, for genomic average nucleotide identity (ANI) analysis (Pritchard *et al.*, 2016). One group is virulent on both cherry and plum and the other only virulent on plum (Hulin *et al.*, 2018a, 2018b). Similarly, *P*. *syringae* pv. *avii* and *P*. *syringae* pv. *persicae* are very closely related but may have host range differences within *Prunus*, with the former being restricted to wild cherry (Ménard *et al.*, 2003). Further rigorous pathogenicity testing will be required to determine the level of host specificity within the other *Prunus*‐infecting clades.

The diversity of bacteria causing canker raises issues of taxonomy and nomenclature, not only for the scientific community, but also practically for development of control measures, plant health regulations, and diagnostics. Whole genome data confirm that the various canker pathogens do indeed fall into separate phylogenomic species based on the established ANI cut‐off of 94%–95% (Gomila *et al.*, 2017). This divergence in their core genome does not necessarily mean that they use distinct mechanisms of pathogenicity (see discussion on shared flexible effector genes below). In terms of classification, the naming of Psm R1 and R2 as *P*. *syringae* pv. *morsprunorum* (Psm) wrongly suggests genetic similarity and thus the use of separate phylogenomic species names *P*. *amygdali* (Psm R1) and *P*. *avellanae* (Psm R2) has been suggested to reflect their phylogenetic positions (Bultreys and Kałużna, 2010; Marcelletti and Scortichini, 2019). By contrast, whole genome comparisons of *P*. *syringae* pv. *avii* and *P*. *syringae* pv. *persicae* show they have an ANI of >99.8% (M. T. Hulin, unpublished observation) and thus should perhaps be named the same.

The combined contributions of phenotypic and/or genomic data in naming these bacteria is open to debate. It is unclear if using the proposed phylogenomic species in Table 1 is helpful for classification, particularly as it retains partial reference to the older pathovar system. For example, by naming phylogroup 3 strains *P*. *amygdali*, do we cause more confusion as it is unlikely all strains cause disease on *Prunus amygdalus* from which the name is derived? In addition, although genome sequencing allows separation of strains into what are sometimes elevated to the rank of new species, for example *P*. *cerasi*, it is debatable whether or not introducing a new species name really helps us to understand the disease in practice. To avoid taxonomic red herrings, one possible solution would be to revert to a pathology‐based nomenclature with all genuine canker‐causing strains being named *P*. *syringae* pv. *cancriprunorum* (i.e., pathovar from the *P*. *syringae* species complex causing canker in *Prunus*). This would allow more focus on and dissection of pathogenicity mechanisms encoded by genes that may be shared requirements for pathogenicity, but are outside the different core genomes. The addition of phylogroup information after this could then add a level of genomic information, for example, *P*. *syringae* pv. *cancriprunorum* phylogroup 1.

## 4 WHAT MAKES A CHERRY PATHOGEN?

How do the different clades of *P*. *syringae* cause bacterial canker? *P*. *syringae* is a model organism in the study of plant–microbe interactions (Mansfield *et al.*, 2012). Pathogenicity factors have been well studied in model pathosystems using tomato, *Arabidopsis*, and bean (Preston, 2000; Arnold *et al.*, 2011). Essential requirements for virulence have been identified, including the production of effector proteins injected into plant cells through the type III secretion system (T3SS), toxins, and phytohormones (reviewed in Caballo‐Ponce *et al.*, 2017; Xin *et al.*, 2018). These factors may act synergistically to overcome plant defences.

The plant immune system can be arbitrarily divided into pathogen/microbe‐associated molecular pattern (PAMP/MAMP)‐triggered immunity (PTI), involving the detection of conserved microbial components such as flagellin, and effector‐triggered immunity (ETI), which is induced by the detection of effector proteins (Jones and Dangl, 2006). Effectors are essential for virulence as they suppress both PTI and ETI, but conversely can activate ETI when resistance (*R*) gene products detect the presence of the type III effector (T3E), directly or indirectly.

To understand how each of the different clades have evolved virulence on cherry, genomic analysis allowed the identification of the sets of genes encoding T3Es and toxin and hormone biosynthesis (Hulin *et al.*, 2018a; Ruinelli *et al.*, 2019; Marcelletti and Scortichini, 2019).

### 4.1 Type III effectors (T3Es)

Despite their common host, cherry‐infecting strains show remarkable variation in their number and composition of T3E repertoires (Hulin *et al.*, 2018a), reflecting their phylogenetic divergence and perhaps the functional redundancy among T3Es (Lindeberg *et al.*, 2012). Extensive research of the model pathogen *P*. *syringae* pv. *tomato* DC3000 has revealed that effectors can be grouped into redundant effector groups (REGs). These contain effectors with overlapping virulence roles (Kvitko *et al.*, 2009; Cunnac *et al.*, 2011). One would hypothesize that the *Prunus*‐infecting clades each possess members of the REGs required for virulence on *Prunus*. Notably however, *P*. *viridiflava* strains from *Prunus* do not always possess the genes encoding the canonical T3SS, and thus the virulence strategy of strains in this clade may differ drastically from the other *Prunus*‐infecting strains, which are within the primary phylogroups of *P*. *syringae* (Dillon *et al.*, 2019).

#### 4.1.1 T3Es promoting virulence?

Using phylogenetically aware association statistics on the cherry pathogens with a canonical T3SS, a set of T3Es was identified that are over‐represented in cherry pathogenic clades, and therefore are putatively of common importance in this disease. This approach identified several candidate virulence‐associated T3E genes: *hopAR1*, *hopBB1*, *hopBF1*, and *hopH1*, which are present in many cherry pathogen genomes across the phylogeny and have probably been gained in these clades.

**HopAR1** (also known as AvrPphB) is a C58 YopT cysteine protease that recognizes and cleaves receptor‐like cytoplasmic protein kinases (RLCKs) such as BIK1, RIPK, PBS1, and various PBS‐like kinases in *Arabidopsis* crucial for both the PTI and ETI response of the plant (Zhang *et al.*, 2010; Russell *et al.*, 2015). HopAR1 has also been shown to cleave homologues of PBS1 in barley (Carter *et al.*, 2019), indicating its targets may be conserved across plant families. We cannot assume it has the same role in cherry without validation by identification of interacting partners, so its function in this host is still to be determined. Members of Psm R1, Psm R2, *P*. *syringae* pv. *avii*, *P*. *cerasi*, and Pss within the subphylogroup 2d possess the *hopAR1* gene. *P*. *syringae* also possesses homologous YopT cysteine proteases, such as HopAY1 and HopAW1, for which the host targets are yet to be characterized and are potentially different from those of HopAR1 (Dowen *et al.*, 2009). Interestingly, many cherry pathogens also possess multiple copies of genes encoding these effector families (Hulin *et al.*, 2018a).

**HopBB1** is part of the HopF family containing multidomain proteins with homologous N‐termini and myristoylation sites for plant cell membrane localization (Lo *et al.*, 2016). HopBB1 is a chimeric effector as its C‐terminus is not homologous to the other family members, which may indicate different functionality. In *Arabidopsis*, HopBB1 specifically interferes with hormonal signalling, promoting jasmonic acid‐activated pathways by the removal of two repressors, TCP14 and JAZ3. This leads to the down‐regulation of salicylic acid signalling, important for defence against *P*. *syringae* (Yang *et al.*, 2017). The manipulation of jasmonic acid signalling to suppress immunity is a role shared by other effectors such as HopX1 and HopZ1a as well as the toxin coronatine (Jiang *et al.*, 2013; Gimenez‐Ibanez *et al.*, 2014). By contrast, other HopF members, such as HopF2, have been shown to interfere with immune signalling through interactions with RIN4, MKK4, and BAK1 (Lo *et al.*, 2016). It is unclear if HopBB1 shares any functional similarities with other HopF members and if its role in manipulation of hormone signalling is conserved in cherry. However, an interesting note is that members of Psm R1, *P*. *syringae* pv. *cerasicola*, and Psm R2 each possess two different alleles of the *hopF* gene as well as the *hopBB1* gene (Hulin *et al.*, 2018a). Such duplications indicate that this effector family may be enriched in cherry pathogens and is therefore a target for further study.

The **HopBF1** effector is a protein kinase that can inactivate the chaperone Hsp90, preventing its role in activating key immune receptors during plant immunity (Lopez *et al.*, 2019). Its representation across the cherry pathogenic clades is intriguing as this effector is relatively rare across the whole *P*. *syringae* phylogeny (18% of all strains compared to 45% of cherry isolates).

The final effector showing evolution correlated with cherry pathogenicity is the protease **HopH1**. Relatively little is known about its role *in planta*, although it has a Zn‐dependent protease motif and is homologous to the *Ralstonia solanacearum* Rip36 protein (Nahar *et al.*, 2014). It forms a REG with HopC1 in *P*. *syringae* pv. *tomato* DC3000 during *Arabidopsis* infection (Wei *et al.*, 2007), indicating a similar role in virulence, and is often clustered with this effector physically on the genome (Baltrus *et al.*, 2012; Newberry *et al.*, 2019). Although the four highlighted effectors appear to have important roles in virulence, further functional characterization is required to support the *in silico* predictions. Not all of these effectors  were encoded by all pathogenic strains, indicating that their functions may sometimes be replaced by other effectors either singly or in combination.

#### 4.1.2 T3Es triggering resistance

In contrast to the positive link between effector presence and virulence, the absence of two T3E families (*avrPto* and *hopAB*) is closely associated with cherry pathogenicity. Alleles of these effectors are either absent from or truncated in cherry‐pathogenic clades. The AvrPto1 and HopAB2 effectors do not share sequence similarity or domains, but their functions in virulence overlap, meaning they form a REG in the model pathosystem of *P*. *syringae* pv. *tomato* DC3000 ∆*hopQ1‐1* on *Nicotiana benthamiana* (Kvitko *et al.*, 2009) and are vital for early suppression of PTI. A related *hopAB* allele, *hopAB1*, is important for pathogenicity of divergent clades, *P*. *syringae* pv. *phaseolicola* and *P*. *syringae* pv. *syringae* on French bean (Jackson *et al.*, 1999; Helmann *et al.*, 2019). As this function is important for disease on these hosts, perhaps other T3Es take on this role in suppression of the cherry immune system? The alternative possibility, that some functions are not required or less important for infection of cherry, should also be considered.

To determine if AvrPto and HopAB trigger ETI in cherry, they were ectopically expressed in cherry‐pathogenic strains in the laboratory (Hulin *et al.*, 2018a). Expression of several alleles of the *hopAB* gene triggered a hypersensitive reaction (HR) in cherry (faster symptom onset at high inoculum concentration) and restricted bacterial multiplication from lower doses *in planta*. The *avrPto1* and *hopAB1* effector genes were found in the clade of Psm R1 plum‐infecting strains that exhibit reduced aggressiveness on cherry. One hypothesis is that selection pressures due to the activation of ETI could have driven the loss of these genes to enable greater virulence on cherry. The truncation of *hopAB3* in Psm R2 and indel in this allele in *P*. *syringae* pv. *avii* could also have been selected during the evolution of these clades to maximize disease on cherry by avoiding recognition of sites in the HopAB effector protein (Hulin *et al.*, 2018a). As HopAB3 is a multidomain protein, the truncated version in Psm R2 may still function and play a role during virulence on cherry. Similar truncated forms of HopAB lacking the E3 ubiquitin ligase domain or with diminished enzymatic activity of this domain have also been identified in other pathovars (Lin *et al.*, 2006; Chien *et al.*, 2013).

Other studies have examined similarly divergent lineages of *P*. *syringae* that infect a common host and identified genomic signatures of host specificity. Newberry *et al. *(2019) used pangenomic analysis of curcubit strains across phylogroup 2, and hypothesized that the T3Es HopC1, HopH1, and HopAR1 may serve as negative pathogenicity factors in cucurbits, whilst HopZ5 may be important for pathogenicity due to its convergent gain across two clades of phylogroup 2. Interestingly, HopC1 expression in cherry strains led to reduced population growth *in planta*, indicating that it may be an avirulence factor that triggers immunity in cherry (Hulin *et al.*, 2018a).

### 4.2 Toxins

Other virulence factors used to manipulate the host include toxins that are not secreted via the T3SS and have been historically identified based on their contributions to symptom development (Bender *et al.*, 1999). Strains of Pss in phylogroup 2 possess several gene clusters for toxin biosynthesis, and also generally have much smaller effector repertoires than other phylogroups; the toxins may compensate for the reduced set of effector proteins (Figure 3). Gene clusters for up to four toxins, syringomycin, syringolin A, syringopeptin, and mangotoxin, are present in Pss strains.

Syringomycin and syringopeptin are produced after detection of plant signal molecules in a similar manner to the T3SS (Wang *et al.*, 2006). Single and double knockouts of genes involved in these pathways revealed that both toxins contribute to symptom development in immature cherry fruits (Scholz‐Schroeder *et al.*, 2001). A study of Pss infecting bean showed that syringomycin may also be required for competitive fitness of bacteria in the plant apoplast (Helmann *et al.*, 2019). The toxins are antagonistic to other bacteria and also fungi, suggesting that they also function in microbial competition (Lavermicocca *et al.*, 1997). By contrast, syringolin A, a nonribosomal cyclic peptide fused with a polyketide synthase, acts within plant cells to inhibit the proteasome interfering with the immune response (Schellenberg *et al.*, 2010). It has also been shown to promote the spread of bacteria by suppressing immune responses in neighbouring plant tissues and increasing bacterial motility (Misas‐Villamil *et al.*, 2013).

Mangotoxin is an antimetabolite toxin present in phylogroup 2a and 2b, which includes some cherry and plum strains. Originally identified in Pss pathogenic to mango, it inhibits the biosynthesis of ornithine and arginine and has been implemented as a virulence factor as it increases disease symptoms and may also improve epiphytic fitness (Arrebola *et al.*, 2009).

Some Psm R1 strains possess the gene clusters for the biosynthesis of coronatine, a chlorosis‐inducing polyketide toxin that interferes with hormone signalling in plant cells to induce stomatal opening and interrupt the salicylic acid‐associated immune response (Grant and Jones, 2009). Coronatine expression is co‐regulated with the T3SS as it is induced in a *hrpL*‐dependent manner in *P*. *syringae* pv. *tomato* DC3000 (Fouts *et al.*, 2002). The possession of coronatine genes is one of several discriminating factors that separate strains of Psm R1 with high and low aggressiveness on cherry (Gilbert *et al.*, 2009; Hulin *et al.*, 2018a).

### 4.3 Hormones

Hormone production is a common trait of plant‐associated bacteria, which can lead to hormonal imbalance and therefore modification of plant growth and development (Aragón *et al.*, 2014). Auxin and cytokinin production have been extensively studied in the gall‐forming olive knot pathogen *P*. *savastanoi* pv. *savastanoi*, in which their production is required for full expression of knot symptoms (see reviews by Ramos *et al.*, 2012 and Caballo‐Ponce *et al.*, 2017). Two key genes involved in auxin (IAA) biosynthesis, *iaaH* and *iaaM*, are present in many pathovars of *P*. *syringae* (Kunkel and Harper, 2018), including *Prunus* pathogens (Hulin *et al.*, 2018a). By contrast, the gene encoding an isopentenyl transferase (*ptz*) required for cytokinin production is not widely distributed within the *P*. *syringae* species complex. Although it is present in the gall‐forming *P*. *syringae* pv. *cerasicola* in phylogroup 3, it is absent from the other pathogens of *Prunus* that principally cause cankers (Ruinelli *et al.*, 2019). Disruption of the phytohormone balance would be expected to lead to the overgrowth associated with canker symptoms but the precise role of IAA and cytokinin production by these pathogens is unclear and remains to be explored.

### 4.4 Diverse weaponry leads to pathogenicity

The differences in the complements of virulence factors between the *Prunus*‐infecting *P*. *syringae* clades may reflect their subtle differences in pathogenicity. In particular, toxin production is probably the major factor that leads to different symptoms on fruits seen in Figure 2 (Scholz‐Schroeder *et al.*, 2001). Cherry leaf inoculations revealed that Pss induces disease symptoms more rapidly than the Psm races, indicating that it may have a more necrotrophic lifestyle (Hulin *et al.*, 2018b). With only a core set of T3Es lacking redundancy, Pss may be less able to suppress the immune system and therefore be unable to remain biotrophic for as long as members of other clades. This idea requires support from further experimental evidence, such as looking at the timing of toxin expression and when the switch to necrotrophy occurs. Continued proliferation of the bacterial population after plant cell death would also provide evidence for necrotrophy alongside symptomology.

The reduced repertoire of T3Es in Pss and other members of phylogroup 2 may also expand the host range of this clade, as they hypothetically possess fewer ETI‐activating effectors. However, a preliminary assessment of different Pss strains isolated from cherry, plum, bean, pea, and lilac inoculated onto detached cherry leaves revealed that the strains from *Prunus* did grow to higher population levels *in planta* than those from other plants, indicating that some host adaptation does exist within phylogroup 2 (Hulin *et al.*, 2018a). A host specificity study on cucurbits showed remarkable variation in the virulence of phylogroup 2 strains on two different host species (Newberry *et al.*, 2019), which was postulated to be due to differences in effector repertoires. Another study by Rezaei and Taghavi (2014) also supports the concept of host specialization within this phylogroup following careful examination of a range of strains on different hosts; a strain of Pss isolated from wheat failed to cause disease symptoms and reached lower bacterial populations in cherry leaves than strains originating from *Prunus*, although all strains multiplied equally well in peach leaves regardless of their host of isolation. Nonetheless, the distribution of cherry‐isolated strains across phylogroup 2 raises questions about the formation of separate populations of these groups and the relative ease with which this phylogroup adapts towards new hosts.

Genomics can generate a list of candidate genes for functional validation in a particular plant‐host system. However, this work highlights the broader challenge of applying knowledge to non‐model systems. In particular, can the function of an effector in cherry pathogens be inferred from homology alone? The situation is complex as effectors, particularly those with multiple domains, have been shown to target numerous host receptors and can act within a hierarchical network dependent on the presence of other effector functions (Wei and Collmer, 2018). Single nucleotide changes within effector genes or their promoters could lead to modified expression, localization, or altered functionality, parameters that cannot currently be modelled deeply in genomic analyses. This may change as the understanding of the link between DNA sequence, protein structure, and protein–protein interactions increases. With more data, and perhaps with the help of machine and deep‐learning approaches, there may eventually be enough power to begin to model and predict effector function from DNA sequence alone (Sperschneider, 2019).

## 5 CHANGING FRUITS: THE ROLE OF HORIZONTAL GENE TRANSFER

What are the origins of factors important for virulence on *Prunus*? An examination of horizontal gene transfer of T3E genes found that transfers were predicted between Psm R1, Psm R2, and *P*. *syringae* pv. *avii* (Hulin *et al.*, 2018a). With the use of long‐read sequencing technologies, recent studies have been able to resolve the complete genomes of cherry pathogens into chromosomal and plasmid sequences (Hulin *et al.*, 2018a; Ruinelli *et al.*, 2019). Complete genomes can greatly improve the accuracy of downstream analyses by resolving highly repetitive regions and providing structural context to gene locations (Baltrus and Clark, 2019; Smits, 2019). Careful re‐examination of the presence of T3E genes in the whole genomes of cherry pathogens characterized by long‐read sequencing technologies has revealed some effector genes are present in multiple copies (authors’ unpublished observation). The *hopAY1* gene is present in two copies in certain complete Psm R1, Psm R2, and *P*. *syringae* pv. *avii* genomes. In addition, two copies each of *hopF3*, *hopBL2*, and *hopAO2* are present in Psm R1 (Table S2) that were unresolved in draft genomes. The significance of these duplications is unclear but they may allow enhanced expression of valuable effectors.

**FIGURE 4:**
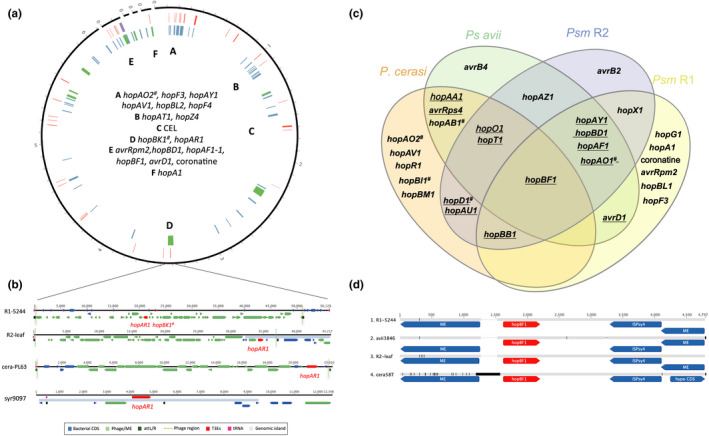
Role of horizontal gene transfer in evolution of cherry pathogens. (a) Circular plot of the complete *Pseudomonas syringae* pv. *morsprunorum* (Psm) R1‐5244 genome created using Circos (Krzywinski *et al.*, 2009). Circles going outwards: predicted prophages (green) and genomic islands (blue), effectors (red), and toxins (purple). Prophage regions and genomic islands identified using PHASTER (Arndt *et al.*, 2016) and IslandViewer 4 (Bertelli *et al.*, 2017), respectively. Example regions of interest where virulence genes are clustered are labelled A–F. # Effector gene is disrupted in some way. (b) Prophage regions carrying the *hopAR1* gene in different *Prunus* pathogens. The key shows gene/region labelling by colour. (c) Venn diagram of the type III effectors (T3Es) carried on plasmids in complete genomes of cherry‐pathogenic *P*. *syringae*. Many T3Es are present among the different pathogen groups and present on plasmids. Underlined effectors showed evidence of horizontal gene transfer between cherry pathogens based on protein phylogenies showing clustering of pathogens incongruent with the core genome phylogeny. # Effector gene is disrupted in some way. (d) Alignment of the region surrounding the *hopBF1* gene in different cherry pathogens showing flanking regions and identical effector sequence. The grey bars show sequence similarity whilst black indicates mismatches

Extending the effector analysis to the recently identified cherry pathogen *P*. *cerasi* shows that it may also share effectors with the other cherry‐infecting clades (Ruinelli *et al.*, 2019; M. T. Hulin, unpublished observation). Many of the shared T3Es are located on plasmids (Figure 4), suggestive of horizontal gene transfer between the cherry pathogens in different phylogroups. Strains within the clades Psm R1, Psm R2, *P*. *cerasi*, and *P*. *syringae* pv. *avii* possess a highly variable number of plasmids (none to seven), with most possessing at least one, whilst Pss strains rarely possess plasmids, ranging from none to two (Liang *et al.*, 1994; Hulin *et al.*, 2018a; Ruinelli *et al.*, 2019). In Psm R1, coronatine has been found to be plasmid‐encoded, suggesting that it has been gained in this clade via horizontal gene transfer (Hulin *et al.*, 2018a). However, the striking lack of plasmids in many Pss strains does indicate that plasmids may not be a major vehicle for gene exchange within this clade.

Examination of other mobile elements (Hulin *et al.*, 2018a) revealed that various T3Es are also present within genomic islands in the different *Prunus*‐infecting clades. Of particular interest is the virulence‐associated T3E gene *hopAR1* that has been convergently acquired in the different clades. This T3E gene is on the chromosome in all clades. It is present within distinct prophage sequences within Psm R1 (closest hit *Pseudomonas* phage JBD25) and Psm R2 (closest hit *Pseudomonas* phage phi3), whilst being on a genomic island in Pss strains. The Psm R2 prophage region is incomplete, suggesting it is unlikely to be active. It is homologous to the region containing the *hopAR1* gene in some phylogroup 2 strains including *P*. *cerasi*, indicating possible past transfer of this T3E between phylogroups via a prophage. In comparison, the Psm R1 prophage carrying *hopAR1* also carries a disrupted version of the *hopBK1* effector and *in silico* predictions suggest it is still functional. Preliminary experiments have confirmed that it is able to excise from the chromosome and circularize (M. T. Hulin, unpublished observation). The convergent acquisition of the *hopAR1* gene is intriguing as it has been independently acquired and retained in different clades, suggesting its importance in virulence. Prophages are known to carry virulence genes in many lysogenic animal pathogens such as *Escherichia coli* (Ohnishi *et al.*, 2001) and *Vibrio cholerae* (Waldor and Mekalanos, 1996); however, this was the first finding of a *P*. *syringae* T3E within a putatively active prophage.

The selective retention of some common T3Es among cherry‐pathogenic clades points to a role in virulence. In addition, some T3Es may be transferred together or hitchhike with functionally important genes due to linkage on plasmids or other mobile genetic elements and therefore could be less important than hypothesized. For example, the *hopH1* gene is within 10 kb of other effector genes such as *hopF4* in Psm R2 (on a putative integrative conjugative element, ICE) and a disrupted copy of the *hopAW1* gene in Pss (Hulin *et al.*, 2018a). Interestingly, Newberry *et al. *(2019) also found the *hopH1* gene within ICEs containing a variety of other effectors such as *hopC1*, *hopAR1*, *hopAW1*, *avrRpt2*, *hopZ1*, and *hopZ5* in curcurbit‐infecting Pss. This gene therefore appears to be moving in close association with other effectors. Dissecting the individual contributions of these co‐gained effectors may be challenging, particularly in the background of effector‐rich strains such as Psm R1 and R2.

## 6 EFFECTORS AND LIFE OUTSIDE THE HOST PLANT

An important caveat in population genomic analyses is that there is an inherent bias towards sampling pathogenic strains of *P*. *syringae* from important crops (discussed in Monteil *et al.*, 2016). This may mean that we are missing the interactions occurring between pathogens and the *P*. *syringae* metapopulation in the wider environment such as stages of the water cycle and during interactions with wild plant species (reviewed in Morris *et al.*, 2013). Even within fruit orchards, sampling has been focused on diseased tissues, giving us a narrow view of the lineages occupying this  environment. More unbiased sampling, including leaf washing and sampling symptomless tissues, has revealed other *P*. *syringae* strains within *Prunus* and kiwifruit orchards that belong to unknown clades and often exhibit low virulence (Vicente *et al.*, 2004; Gilbert *et al.*, 2008, 2009; Straub *et al.*, 2018; Visnovsky *et al.*, 2019).

It appears that the major pathogens often coexist with a diverse range of other clades. More systematic sampling designs are required to characterize the bacterial populations inhabiting orchard environments, determine the genetic interactions occurring, and identify the factors that differentiate pathogenic lineages from the wider population. It is likely that epiphytic strains within *Prunus* orchards exhibit a spectrum of virulence. Subpopulations may possess factors that provide a basal level of pathogenicity such as the T3SS. For example, a study of the endophytic growth of a variety of strains on kiwifruit (Bartoli *et al.*, 2015) revealed that a set of genes encoding the catechol degradation pathway allowed increased colonization of internal plant tissues, a step towards the evolution of a pathogenic/endophytic lifestyle in populations adapted to surface conditions. Interactions between members of *P*. *syringae* on the plant surface could eventually lead to newly virulent lineages emerging through recombination.

## 7 HOW TO INFECT WOODY TISSUES

The ability to colonize and cause disease in trees has evolved multiple times within the *P*. *syringae* complex (Nowell *et al.*, 2016). Woody tissues are so heterogeneous, comprising living cambium and dead lignified vessels, it is difficult to define precisely what special factors, if any, may be required for their colonization. However, once inside a cherry branch for example, bacteria are within an environment rich in available nutrients and protected from adverse conditions. The symptomology associated with *P*. *syringae* diseases in wood varies considerably between host plants and *P*. *syringae* clades and reflects dramatic host responses as well as pathogen‐induced necroses (Lamichhane *et al.*, 2014). The term canker is defined as a necrotic, usually sunken, lesion on the woody tissue of a plant (Agrios, 1936) and is a symptom of various bacterial and fungal diseases. It is usually associated with gummosis and dieback. Canker symptoms are common in many tree hosts infected with *P*. *syringae* other than *Prunus*, including horse chestnut, kiwifruit, and hazelnut (Schmidt *et al.*, 2008; Ferrante and Scortichini, 2010; Scortichini, 2010). Infection of actively growing shoot tips is also associated with necrosis, referred to as shoot dieback or when rapid, shoot blight/blast (Wimalajeewa, 1987). Blister bark is a symptom seen during Pss infection of apple, whereby the tissue becomes raised, flaky, and necrotic (Scortichini and Morone, 1997). Finally, certain clades within phylogroup 3 cause tumour‐like galls as seen in bacterial gall of ornamental *Prunus* (*P*. *syringae* pv. *cerasicola*) and olive knot disease (*P*. *syringae* pv. *savastanoi*) (Kamiunten *et al.*, 2000; Lamichhane *et al.*, 2014).

Clades of *P*. *syringae* causing tree diseases may not contain the battery of plant cell wall‐degrading enzymes allowing lignin degradation per se. However, a gene cluster involved in the metabolism of aromatic compounds (designated WHOP, woody, hosts and *Pseudomonas*) (Rodríguez‐Palenzuela *et al.*, 2010; Caballo‐Ponce *et al.*, 2016) is found in several wood‐infecting strains. This 15 kb genomic region contains sets of genes involved in phenolic (lignin) metabolism, such as the breakdown of anthranilate to catechol (*antABC*) and the subsequent degradation of catechol (*catBCA*). The cluster may allow the degradation of potentially antibacterial lignin precursors. Deletion of these genes showed that they are important in the aggressiveness of *P*. *syringae* pv. *savastanoi* on woody olive tissue (Caballo‐Ponce *et al.*, 2016). Bartoli *et al. *(2015) determined that catechol degradation via the β‐ketoadipate pathway was correlated with ability to survive endophytically and cause disease symptoms on kiwifruit. The WHOP gene cluster is present in the phylogroup 3 subclade containing both Psm R1 and *P*. *syringae* pv. *cerasicola*. It is also present in Psm R2 and its close relatives and is partially present in some *Prunus*‐infecting Pss strains (Hulin *et al.*, 2018a). It may therefore have a role in bacterial fitness within *Prunus* woody tissues. However, this gene cluster is absent from other clades that cause canker on *Prunus*, such as *P*. *syringae* pv. *avii*, *P*. *syringae* pv. *persicae*, *P*. *cerasi* and the majority of Pss strains (Hulin *et al.*, 2018a). This absence indicates that it is either not essential for virulence or that other unidentified genes are fulfilling the same role in other clades. Studies of the horse chestnut and kiwifruit pathogens (Green *et al.*, 2010; Cunty *et al.*, 2015) have revealed that strains vary in ability to invade leaf or woody tissues and similar experiments are needed to assess the significance of wood colonization in *Prunus*.

Nowell *et al. *(2016) also found that several effector genes such as *hopAY1* and *hopAO1* were enriched in clades of woody plant‐infecting pathovars. The role of T3Es during woody tissue infection has been shown in olive where the T3SS is essential for growth of *P*. *syringae* pv. *savastanoi* (Caballo‐Ponce *et al.*, 2016). Tissue‐specific roles of T3Es have yet to be found in *P*. *syringae*, although varying resistance responses can occur between tissues (Tahir *et al.*, 2019).

Canker development in *Prunus* species occurs when the tissue is dormant or just coming out of dormancy and there is extensive lignification that the bacteria must overcome or avoid (Caballo‐Ponce *et al.*, 2017). The bacteria colonize various tissues such as the cortex, cambium, phloem, and xylem (Crosse, 1956; Lamichhane *et al.*, 2014). In the growing season, the other stages of the disease such as shoot, fruit, flower, and leaf necrosis, occur in actively growing nonlignified tissue (Crosse, 1966). In‐depth studies of bacterial gene expression in‐different tissues could provide some insights into whether colonization requires the induction of distinct mechanisms of virulence (Yu *et al.*, 2013). This could be coupled with the identification of mutants deficient in growth and/or symptoms in the different tissues (Somlyai *et al.*, 1986). A recent high‐throughput approach called transposon insertion sequencing (TnSeq) involves inoculating pools of transposon mutants and using sequencing to determine mutant frequencies within this pool before and after exposure to a selective environment, such as within a plant (van Opijnen *et al.*, 2009). This approach has proved powerful in identifying different genes important for bacterial fitness in the apoplast versus surface of leaves in the Pss–bean interaction (Helmann *et al.*, 2019). It could be applied to the *Pseudomonas–Prunus* system to identify candidate virulence genes through an unbiased genetic dissection (Phan *et al.*, 2013).

## 8 TOWARDS VARIETAL RESISTANCE

There are no examples of cherry cultivars with complete immunity or single gene‐based resistance to canker. Broad‐acting partial resistance to all clades exists in certain cultivars, notably Merton Glory (Hulin *et al.*, 2018b). Perennial plants provide challenging study systems as differences in tissue‐specific resistance, plant age, seasonal environment conditions, and nutrient status impact on susceptibility (Kus *et al.*, 2002; Mur *et al.*, 2017; Velásquez *et al.*, 2018; Tahir *et al.*, 2019).

Historical studies have provided clues to responses involved in canker resistance. Timing of leaf‐drop may have a major effect on field resistance as the leaf scar provides a route to infection, particularly in Psm infections. In addition, the timing of break from dormancy and the generation of active phellogen, the meristematic cell layer that generates periderm, which is thought to provide summer immunity, may vary between different cultivars. Varieties that bloom earlier also show reduced canker spread (Wilson, 1939; Crosse, 1966). Since these early studies, little work has been done to identify the immune responses that occur in woody tissues. It is likely that immunity involves the creation of antimicrobial barriers in the cambium, and phellogen activity to seal off and prevent the spread of bacterial infections (Rioux, 1996; Abe *et al.*, 2007). Quantitative differences in these responses between host genotypes may contribute to varietal resistance. The age of the tree and rootstock choice may also have effects on resistance levels due to differences in vigour (Garrett, 1979, 1981; Santi *et al.*, 2004). Analysis of leaf populations revealed that different cultivars may support different levels of epiphytic bacteria. As these epiphytic populations are the major inoculum source, any genetic differences that reduce the growth of aggressive pathogens on the plant surface may also be important in the field (Crosse, 1966).

Although a range of resistance assays in the field and laboratory (Figure 2) distinguish high and low virulence strains, the pathogens often show a large degree of variability in their aggressiveness, particularly after field inoculations. This meant that any clade‐specific trends in resistance were not statistically significant in the experiments described in Hulin *et al. *(2018b). For field inoculations, multiyear, multisite experiments using a variety of *P*. *syringae* strains may be required to fully assess canker resistance. Genomics and functional studies have revealed that cherry mounts a nonhost ETI response towards particular effectors (Hulin *et al.*, 2018a). Small additive ETI‐regulated responses may contribute to varietal resistance and could be clade‐specific.

The use of plant genomic association techniques to identify genes for resistance is increasing. Recently, an association study (Omrani *et al.*, 2019) mapped partial resistance towards Pss in apricot to several quantitative trait loci (QTLs), with two loci explaining 41% and 26% of phenotypic variation, respectively. The candidate loci contained genes annotated as producing products involved in phytohormone signalling, specifically abscisic acid, and may mediate crosstalk between different signalling pathways during immunity. Similarly, another association study of kiwifruit canker resistance identified candidate genes within QTLs involved in PTI. The candidate genes identified are involved in cell wall metabolism and signalling (Tahir *et al.*, 2019). Interestingly, this study used multiple pathogenicity assays to identify resistance QTLs that were tissue‐specific and associated with particular disease symptomology. An approach like this would be highly relevant to *Prunus* canker as it is also a multiple tissue disease.

## 9 LINKING GENOMICS TO THE CONTROL OF CANKER

The wealth of historical and current research on bacterial canker endeavours to provide applied outcomes that can help to control this disease. Pathogen population studies have revealed the sheer complexity of the causal organisms and have led to the development of rapid diagnostic techniques to detect the presence of pathogens and assess their disease potential. Once isolated, strains can be easily discriminated in the laboratory by repetitive extragenic palindromic elements (REP) PCR, toxin‐specific PCRs, and rapid pathogenicity tests on immature cherry fruits (Vicente and Roberts, 2007; Bultreys and Kałużna, 2010). A sensitive, culture‐free technique using real‐time PCR on infected plant material was developed by Kałużna *et al. *(2016a). Based on variability in genomic DNA regions, this PCR specifically differentiates Psm R1 and Psm R2. Members of Pss can be readily differentiated from other clades based on the presence of syringomycin biosynthesis genes (Bultreys and Gheysen, 1999). Specific PCR or loop‐mediated isothermal amplification (LAMP) methods for detection of the other canker pathogens are yet to be developed, but could be designed based on genomic sequence variability for rapid identification (such as for *P*. *syringae* pv. *actinidiae* in Ruinelli *et al.*, 2016).

It is likely that future technologies will aid bacterial canker monitoring and diagnosis. For example, with the ever‐reducing costs of DNA sequencing, future diagnostics methods should involve rapid sequencing and bioinformatics to gauge if the bacterial populations occupying orchards are capable of causing disease and if they are resistant to biocides. This technology is already being used in medicine to identify the causes of epidemics in hospitals and determine antibiotic resistance profiles to inform decision‐making (Jeukens *et al.*, 2019). Such precision diagnostics should be applied in the horticultural industry as it would inform the ideal timing and composition of spray routines. One fast‐growing field of research and development is the use of strain‐specific bacteriophages to control diseases (reviewed in Svircev *et al.*, 2018). The combination of using advanced genomics‐based diagnostics with strain‐specific bacteriophages would be a precise alternative to current control measures for canker, which involve the blanket spraying of copper‐based products.

Given the close association between the presence of certain effectors and virulence (although their importance is still to be examined *in planta*), a simplistic outcome of the genomic analysis is to propose that expression of the matching *R* genes in cherry could confer ETI (Kennelly *et al.*, 2007). For example, the R proteins RPS5 and ZAR1 are known to detect members of the HopAR1 and HopF effector families, respectively (Laflamme *et al.*, 2020) that are common in cherry‐infecting strains. The cherry genome sequence (Shirasawa *et al.*, 2017) could be searched for homologues of these *R* genes and other genes required for effector recognition that have been extensively characterized in model plants. A transgenic approach to stack multiple known *R* genes against the common cherry pathogen effector set would provide a proof of concept for such approaches. Although the idea is promising it would probably be challenging to transfer genes across plant genera and families and maintain the correct functionality whilst not triggering autoimmunity (Zhang and Coaker, 2017). It has been shown to work experimentally in some cases; for example, the transfer of the *RPS4* and *RRS1* genes, involved in effector recognition, from *Arabidopsis* into tomato conferred resistance to several pathogens including *P*. *syringae* pv. *tomato* (Narusaka *et al.*, 2013).

An additional molecular approach to accelerate the search for *R* gene‐mediated resistance would be to utilise effectoromics, which involves screening single or combinations of effectors for their ability to elicit immunity *in planta*. In this targeted approach the wider *Prunus* germplasm, which is potentially amenable to conventional plant breeding into cherry, could be screened for responses to core effectors. Effectoromics has been utilized in wild relatives of potato to identify novel resistance sources to the oomycete pathogen *Phytophthora infestans* (Vleeshouwers *et al.*, 2008). If multiple distinct sources of resistance to one or more clades of *P*. *syringae* could be identified, the underlying resistance genes could be pyramided to provide durable resistance to the canker pathogens. In addition, genomics provides a route to understanding the redundancy and mobility of virulence factors and therefore could be used to predict and monitor virulence factor diversity (Thilliez *et al.*, 2019) and predict the likelihood of resistance failing, due to the loss, modification, or pseudogenization of targeted effectors.

## 10 FUTURE PROSPECTS

Bacterial canker of *Prunus* species is a highly complex disease. Perennial plants provide multiple niches for bacterial occupation including different tissues and host life stages. Progress towards the development of resistant cultivars is impeded by the diversity of bacteria that cause the disease. It is clear that further research into the fundamental mechanisms of resistance and underlying genetics will be beneficial to inform breeding programmes. The diversity of the bacteria associated with the canker syndrome presents a challenge that breeders must appreciate, and strain selection for phenotyping will be key. There has been valuable progress in the development of tools to diagnose the cause of the disease and this will be further enhanced by novel sequencing‐based technologies that could allow rapid in‐field diagnostics and monitoring of disease potential and host range. With a greater knowledge of pathogen lifestyle and genetics, control measures can be made more precise and durable by targeting the particular pathogens that occupy particular tissue types in each orchard during the annual cycle of tree production.

### 10.1 Targets of future research

- A revision of the taxonomy of canker pathogens of *Prunus* would be beneficial.
- Any newly identified pathogens should be examined thoroughly for virulence and host range to assess their possible impact on their species of origin and wider *Prunus*.
- Genetic dissection of the role and regulation of virulence factors such as T3Es and toxins in the colonization of woody tissues will provide insight into niche specialization.
- Further investigation of epiphytic populations on hosts and nonhosts that may act as reservoirs for the pathogens and facilitate gene exchange.
- The identification of the genetic basis of resistance to canker in related *Prunus*.

## CONFLICT OF INTEREST

The authors declare no conflict of interest.

## Supporting information

Table S1

Table S2

Table S3

Text S1

## Data Availability

Data sharing is not applicable to this article as no new data were created or analysed in this study.
