# Supplementary material for: Cherry picking by pseudomonads: After a century of research on canker, genomics provides insights into the evolution of pathogenicity towards stone fruits
Source: Plant Pathol. 2020 May 6;69(6):962–78. doi: 10.1111/ppa.13189 (PMC7386918; doi:10.1111/ppa.13189)
Supplement: Supplementary file 1 — Table S1 [file PPA-69-962-s001.docx]

Table S1: Genome assemblies utilised to create phylogenetic tree in Fig.3 with relevant information and reference

| **Strain** | **Clade** | **Assembly** | **Size (mb)** | **Accession** | **Scaffolds** | **Proteins** | **Level** | **Reference** |
| --- | --- | --- | --- | --- | --- | --- | --- | --- |
| *Pseudomonas syringae* pv. *syringae* B728a | 19543 | GCA_000012245.1 | 6.09 | CP000075 | 1 | 5089 | Complete Genome | Feil *et al.*, 2005 |
| *Pseudomonas syringae* pv. *tomato* str. DC3000 | 19543 | GCA_000007805.1 | 6.54 | AE016853- AE016855 | 3 | 5619 | Complete Genome | Buell *et al.*, 2003 |
| *Pseudomonas* *savastanoi* pv. *phaseolicola* 1448A | 19543 | GCA_000012205.1 | 6.11 | CP000058- CP000060 | 3 | 5165 | Complete Genome | Joardar *et al.*, 2005 |
| *Pseudomonas* *coronafaciens* pv. *porri* LMG 28495 | 19543 | GCA_001275725.1 | 6.01 | JTHM01 | 274 | 5295 | Contig | Rombouts *et al.*, 2016 |
| *Pseudomonas* *amygdali* pv. *lachrymans* NM002 | 19543 | GCA_002068135.1 | 6.01 | CP020351 | 1 | 5375 | Complete Genome | Li *et al.*, 2019 |
| *Pseudomonas* *viridiflava* CFBP1590 | 19543 | GCA_900184295.1 | 6.09 | LT855380 | 1 | 5313 | Complete Genome | Ruinelli *et al.*, 2017 |
| *Pseudomonas* *avellanae* R2leaf | 19543 | GCA_002905795.2 | 6.58 | CP026562-CP0265627 | 6 | 5777 | Complete Genome | Hulin *et al.* 2018 |
| *Pseudomonas* *marginalis* ICMP11289 | 19543 | GCA_001467325.1 | 6.09 | LKGX01 | 134 | 5336 | Contig | Visnovsky *et al.*, 2016 |
| *Pseudomonas* *cannabina* ICMP2823 | 19543 | GCA_900100365.1 | 6.39 | FNKU01 | 9 | 5451 | Contig | Varghese 2016 (ds) |
| *Pseudomonas* *cichorii* JBC1 | 19543 | GCA_000517305.1 | 5.99 | CP007039 | 1 | 5006 | Complete Genome | Ramkumar *et al.*, 2015 |
| *Pseudomonas syringae* pv. *coryli* str. NCPPB4273 | 19543 | GCA_000972175.1 | 6.10 | AWQP01 | 93 | 5305 | Contig | Marcelletti & Scortichini, 2014 |
| *Pseudomonas* *caricapapayae* ICMP2855 | 19543 | GCA_001400735.1 | 6.26 | LJPW01 | 193 | 5560 | Scaffold | Thakur *et al.*, 2016 |
| *Pseudomonas* *tremae* ICMP9151 | 19543 | GCA_001401155.1 | 5.93 | LJRO01 | 510 | 5103 | Scaffold | Thakur *et al.*, 2016 |
| *Pseudomonas syringae* pv. *helianthi* ICMP4531 | 19543 | GCA_001400835.1 | 6.21 | LJQM01 | 277 | 5405 | Scaffold | Thakur *et al.*, 2016 |
| *Pseudomonas* *cerasi* PL58T | 19543 | GCA_900074915.1 | 6.35 | LT222313- LT222319 | 7 | 5478 | Complete Genome | Kałużna *et al.*, 2016 |
| *Pseudomonas* *asturiensis* LMG26898 | 19543 | GCA_900143095.1 | 6.17 | FRDA01 | 42 | 5418 | Scaffold | Varghese 2016 (ds) |
| *Pseudomonas* *caspiana* FBF102 | 19543 | GCA_002158995.1 | 6.10 | LOHF01 | 49 | 5324 | Scaffold | Busquets *et al.*, 2017 |
| *Pseudomonas* *floridensis* GEV388 | 19543 | GCA_002087235.1 | 6.10 | MUIO01 | 287 | 5356 | Scaffold | Timilsina *et al.*, 2018 |
| *Pseudomonas* *graminis* PDD-13b-3 | 19543 | GCA_002093745.1 | 5.69 | MTSB01 | 42 | 4917 | Contig | Besaury *et al.*, 2017 |
| *Pseudomonas syringae* pv. *tagetis* ICMP4091 | 19543 | GCA_001401315.1 | 6.04 | LJRM01 | 208 | 5297 | Scaffold | Thakur *et al.*, 2016 |
| *Pseudomonas* *congelans* RMX815.1a | 19543 | GCA_002318645.1 | 5.57 | NQXZ01 | 167 | 4467 | Contig | Karasov *et al.*, 2017 |
| *Pseudomonas* *avellanae* BPIC 631 | 19543 | GCA_000302915.1 | 6.63 | AKBS01 | 297 | 4994 | Scaffold | O’Brien *et al.*, 2012 |
| *Pseudomonas* *viridiflava* TA043 | 19543 | GCA_000452485.1 | 5.98 | AVDV01 | 218 | 5203 | Scaffold | Baltrus *et al.*, 2014b |
| *Pseudomonas* *amygdali* pv. *morsprunorum* R15244 | 19543 | GCA_002905685.2 | 6.45 | CP026557- CP026561 | 5 | 5643 | Complete Genome | Hulin *et al.* 2018 |
| *Pseudomonas* *coronafaciens* pv. *porri* LMG28496 | 19543 | GCA_001275735.1 | 6.24 | JUEU01 | 369 | 5486 | Contig | Rombouts *et al.*, 2016 |
| *Pseudomonas* *savastanoi* pv. *savastanoi* 3335 | 19543 | GCA_000164015.3 | 6.02 | CP008742 | 1 | 5139 | Complete Genome | Rodríguez-Palenzuela *et al.*, 2010 |
| *Pseudomonas syringae* pv. *tomato* B13-200 | 19543 | GCA_002966555.1 | 6.52 | CP019871-CP019874 | 4 | 5984 | Complete Genome | Xu and Toussaint, 2018 (ds) |
| *Pseudomonas syringae* pv. *actinidiae* ICMP18708 | 19543 | GCA_000344355.2 | 6.63 | CP012179-CP012180 | 2 | 5760 | Complete Genome | Butler *et al.*, 2013 |
| *Pseudomonas syringae* group genomosp. 3 6411 | 19543 | GCA_900235905.1 | 6.36 | LT963408 | 1 | 5628 | Complete Genome | Ruinelli *et al.*, 2019 |
| *Pseudomonas* *coronafaciens* pv. *oryzae* 36_1 | 19543 | GCA_001293935.1 | 5.50 | LGKZ01 | 15 | 4844 | Scaffold | Mott *et al.*, 2016 |
| *Pseudomonas* *amygdali* pv. *aesculi* str. 0893_23 | 19543 | GCA_000145685.1 | 6.01 | AEAD01 | 463 | 5031 | Scaffold | Baltrus *et al.*, 2011 |
| *Pseudomonas* *viridiflava* CC1582 | 19543 | GCA_000452505.1 | 6.02 | AVDW01 | 211 | 5266 | Scaffold | Baltrus *et al.*, 2014b |
| *Pseudomonas* *congelans* NL.P123 | 19543 | GCA_002318745.1 | 5.86 | NQYA01 | 220 | 5030 | Contig | Karasov *et al.*, 2014 |
| *Pseudomonas* *congelans* DM2.1.12.02A | 19543 | GCA_002318885.1 | 5.91 | NQYB01 | 157 | 4848 | Contig | Karasov *et al.*, 2014 |
| *Pseudomonas* *viridiflava* CDRTc14 | 19543 | GCA_001716855.1 | 5.96 | MBPF01 | 38 | 5245 | Scaffold | Samad *et al.*, 2016 |
| *Pseudomonas* *coronafaciens* pv. *garcae* ICMP4323 | 19543 | GCA_001400345.1 | 5.51 | LJQK01 | 109 | 4824 | Scaffold | Thakur *et al.*, 2016 |
| *Pseudomonas* *savastanoi* pv. *glycinea* str. B076 | 19543 | GCA_000187045.2 | 6.24 | AEGG01 | 104 | 5410 | Contig | Qi *et al.*, 2011 |
| *Pseudomonas syringae* CC1557 | 19543 | GCA_000452705.3 | 5.81 | CP007014-CP007015 | 2 | 5000 | Complete Genome | Hockett *et al.*, 2014 |
| *Pseudomonas syringae* pv. *tomato* NYS-T1 | 19543 | GCA_000765305.1 | 6.26 | JRRA01 | 84 | 5690 | Scaffold | Jones *et al.*, 2015 |
| *Pseudomonas* *savastanoi* pv. *glycinea* str. race 4 | 19543 | GCA_000187065.2 | 5.91 | AEGH01 | 108 | 5075 | Contig | Baltrus *et al.*, 2011 |
| *Pseudomonas* *coronafaciens* pv. *atropurpurea* ICMP4457 | 19543 | GCA_001400695.1 | 5.79 | LJPS01 | 167 | 5055 | Scaffold | Thakur *et al.*, 2016 |
| *Pseudomonas* *congelans* ME812.2b | 19543 | GCA_002323035.1 | 5.64 | NQXY01 | 334 | 4861 | Contig | Karasov *et al.*, 2014 |
| *Pseudomonas* *viridiflava* LMCA8 | 19543 | GCA_000834695.1 | 5.99 | JXQO01 | 73 | 5226 | Contig | Perisin 2017 (ds) |
| *Pseudomonas* *savastanoi* pv. *savastanoi* DAPP-PG722 | 19543 | GCA_000732035.1 | 6.42 | JOJV01 | 412 | 5545 | Contig | Moretti *et al.*, 2014 |
| *Pseudomonas syringae* pv. *syringae* HS191 | 19543 | GCA_000988395.1 | 6.00 | CP006256-CP006257 | 2 | 5127 | Complete Genome | Ravindran *et al.*, 2015 |
| *Pseudomonas syringae* pv. *syringae* B301D | 19543 | GCA_000988485.1 | 6.09 | CP005969 | 1 | 5167 | Complete Genome | Ravindran *et al.*, 2015 |
| *Pseudomonas* *congelans* DSM14939 | 19543 | GCA_900103225.1 | 5.74 | FNJH01 | 23 | 5020 | Scaffold | Baltrus *et al.* 2019 (ds) |
| *Pseudomonas* *viridiflava* ICMP13104 | 19543 | GCA_001466975.1 | 5.55 | LKEJ01 | 204 | 4479 | Contig | Visnovsky *et al.*, 2016 |
| *Pseudomonas* *coronafaciens* pv. *zizaniae* ICMP8921 | 19543 | GCA_001401235.1 | 5.73 | LJRT01 | 152 | 5001 | Scaffold | Thakur *et al.*, 2016 |
| *Pseudomonas* *savastanoi* pv. *savastanoi* PseNe107 | 19543 | GCA_000935695.1 | 6.07 | JYHF01 | 247 | 5263 | Contig | Bartoli *et al.*, 2015 |
| *Pseudomonas syringae* pv. *tomato* Max13 | 19543 | GCA_000177475.1 | 6.11 | ADFZ01 | 349 | 5522 | Contig | Vinatzer, 2010 (ds) |
| *Pseudomonas syringae* UMAF0158 | 19543 | GCA_001281365.1 | 5.85 | CP005970-CP005971 | 2 | 5054 | Complete Genome | Martínez-García *et al.*, 2015 |
| *Pseudomonas syringae* pv. *lapsa* ATCC10859 | 19543 | GCA_001482725.1 | 5.92 | CP013183 | 1 | 5052 | Complete Genome | Kong *et al.*, 2016 |
| *Pseudomonas syringae* pv. *tomato* NCPPB1108 | 19543 | GCA_000177495.1 | 6.08 | ADGA01 | 304 | 5450 | Contig | Vinatzer, 2010 (ds) |
| *Pseudomonas* *savastanoi* pv. *nerii* CFBP 5067 | 19543 | GCA_001535815.1 | 5.79 | LIHX01 | 242 | 4965 | Contig | Nowell *et al.*, 2016 |
| *Pseudomonas* *amygdali* pv. *aesculi* str. 2250 | 19543 | GCA_000163275.1 | 5.99 | ACXT01 | 364 | 5038 | Scaffold | Green *et al.*, 2010 |
| *Pseudomonas* *viridiflava* CH409 | 19543 | GCA_002406485.1 | 5.92 | NXDO01 | 80 | 5146 | Contig | Haney *et al.*, 2018 |
| *Pseudomonas* *viridiflava* ICMP8820 | 19543 | GCA_002723575.1 | 5.81 | LKCA01 | 41 | 5081 | Contig | Visnovsky *et al.*, 2016 |
| *Pseudomonas* *savastanoi* pv. *fraxini* CFBP5062 | 19543 | GCA_001538155.1 | 6.26 | LIIC01 | 330 | 5430 | Contig | Nowell *et al.*, 2016 |
| *Pseudomonas syringae* pv. *viburni* CFBP1702 | 19543 | GCA_000935765.1 | 6.49 | JYHK01 | 265 | 5648 | Contig | Bartoli *et al.*, 2015 |
| *Pseudomonas* *amygdali* pv. *tabaci* str. 6605 | 19543 | GCA_000275945.1 | 6.16 | AJXI01 | 283 | 5206 | Contig | Studholme *et al.* 2010 (ds) |
| *Pseudomonas* *avellanae* MAFF212061 | 19543 | GCA_002261495.1 | 7.00 | NKQU01 | 687 | 5245 | Scaffold | Fujikawa & Sawada, 2016 |
| *Pseudomonas* *viridiflava* ICMP2848 | 19543 | GCA_001642795.1 | 5.92 | LKEH01 | 48 | 5179 | Scaffold | Thakur *et al.*, 2016 |
| *Pseudomonas* *amygdali* pv. *tabaci* yuexi-1 | 19543 | GCA_000934645.1 | 6.23 | JWJF01 | 106 | 5326 | Contig | Zhao *et al.* 2014 (ds) |
| *Pseudomonas* *savastanoi* 4352 | 19543 | GCA_001293725.1 | 6.06 | LGKR01 | 121 | 5197 | Scaffold | Mott *et al.*, 2016 |
| *Pseudomonas syringae* pv. *actinidiae* CRAFRU12.29 | 19543 | GCA_002024285.1 | 6.62 | CP019730-CP019731 | 2 | 5726 | Complete Genome | Firrao *et al.*, 2018 |
| *Pseudomonas* *savastanoi* pv. *phaseolicola* HB10Y | 19543 | GCA_001294025.1 | 6.15 | LGKY01 | 96 | 5211 | Scaffold | Mott *et al.*, 2016 |
| *Pseudomonas syringae* pv. *actinidiae* MAFF212063 | 19543 | GCA_002763655.1 | 6.69 | CP024712- CP024714 | 3 | 5776 | Complete Genome | Poulter *et al.*, 2017 |
| *Pseudomonas syringae* pv. *syringae* Pss9097 | 19543 | GCA_002905815.2 | 5.93 | CP026568 | 1 | 5014 | Complete Genome | Hulin *et al.* 2018 |
| *Pseudomonas* *savastanoi* pv. *phaseolicola* HB10Y | 19543 | GCA_001294035.1 | 5.94 | LGKX01 | 44 | 5024 | Scaffold | Thakur *et al.*, 2016 |
| *Pseudomonas* *amygdali* pv. *morsprunorum* 2341 | 19543 | GCA_001535735.1 | 6.28 | LIIB01 | 173 | 5553 | Contig | Nowell *et al.*, 2016 |
| *Pseudomonas* *savastanoi* pv. *phaseolicola* Y5_2 | 19543 | GCA_001294065.1 | 6.21 | LGKV01 | 50 | 4953 | Scaffold | Thakur *et al.*, 2016 |
| *Pseudomonas syringae* pv. *atrofaciens* LMG5095 | 19543 | GCA_003047185.1 | 6.08 | CP028490 | 1 | 5140 | Complete Genome | Cha *et al.* 2018 |
| *Pseudomonas syringae* CFBP3840 | 19543 | GCA_900235815.1 | 6.38 | LT963409-LT963413 | 5 | 5625 | Complete Genome | Ruinelli *et al.*, 2019 |
| *Pseudomonas syringae* group genomosp. 3 9643 | 19543 | GCA_002906035.1 | 5.94 | MLET01 | 58 | 5353 | Contig | Hulin *et al.* 2018 |
| *Pseudomonas* *savastanoi* pv. *phaseolicola* NPS3121 | 19543 | GCA_001294105.1 | 5.99 | LGKW01 | 70 | 4980 | Scaffold | Thakur *et al.*, 2016 |
| *Pseudomonas* *savastanoi* pv. *glycinea* KN44 | 19543 | GCA_001294195.1 | 6.06 | LGLP01 | 215 | 5081 | Scaffold | Thakur *et al.*, 2016 |
| *Pseudomonas syringae* group genomosp. 3 RM1 | 19543 | GCA_002906055.1 | 6.31 | MLEU01 | 95 | 5600 | Contig | Hulin *et al.* 2018 |
| *Pseudomonas syringae* pv. *syringae* CFBP4215 | 19543 | GCA_900235825.1 | 6.04 | LT962480 | 1 | 5126 | Complete Genome | Ruinelli *et al.*, 2019 |
| *Pseudomonas syringae* pv. *avii* CFBP3846 | 19543 | GCA_900235835.1 | 6.50 | LT963402-LT963407 | 6 | 5696 | Complete Genome | Ruinelli *et al.*, 2019 |
| *Pseudomonas* *savastanoi* pv. *glycinea* BR1 | 19543 | GCA_001294265.1 | 6.17 | LGLO01 | 127 | 5175 | Scaffold | Thakur *et al.*, 2016 |
| *Pseudomonas* *amygdali* pv. *myricae* CFBP2897 | 19543 | GCA_001535805.1 | 6.03 | LIHY01 | 203 | 5189 | Contig | Nowell *et al.*, 2016 |
| *Pseudomonas* *amygdali* pv. *ulmi* CFBP1407 | 19543 | GCA_001535915.1 | 6.40 | LIHQ01 | 319 | 5498 | Contig | Nowell *et al.*, 2016 |
| *Pseudomonas* *savastanoi* pv. *glycinea* UnB647 | 19543 | GCA_001294275.1 | 6.05 | LGLL01 | 144 | 5068 | Scaffold | Thakur *et al.*, 2016 |
| *Pseudomonas syringae* pv. *persicae* CFBP1573P1 | 19543 | GCA_900235855.1 | 6.46 | ODAL01 | 214 | 5666 | Contig | Ruinelli *et al.*, 2019 |
| *Pseudomonas syringae* pv. *syringae* CFBP2118 | 19543 | GCA_900235865.1 | 6.03 | LT962481 | 1 | 5121 | Complete Genome | Ruinelli *et al.*, 2019 |
| *Pseudomonas syringae* group genomosp. 3 CFBP3800 | 19543 | GCA_900289095.1 | 6.45 | OLMQ01 | 3 | 5673 | Contig | Ruinelli *et al.*, 2019 |
| *Pseudomonas syringae* pv. *maculicola* CFBP1657 | 19543 | GCA_000935725.1 | 6.06 | JYHH01 | 138 | 5283 | Scaffold | Bartoli *et al.*, 2015 |
| *Pseudomonas syringae* CFBP2116 | 19543 | GCA_900289125.1 | 6.26 | LT985192- LT985195 | 4 | 5551 | Complete Genome | Ruinelli *et al.*, 2019 |
| *Pseudomonas syringae* 31R1 | 19543 | GCA_900105295.1 | 5.87 | LT629769 | 1 | 5056 | Chromosome | Varghese 2016 (ds) |
| *Pseudomonas syringae* pv. *maculicola* YM7930 | 19543 | GCA_001293575.1 | 5.91 | LGLD01 | 64 | 5072 | Scaffold | Mott *et al.*, 2016 |
| *Pseudomonas* *savastanoi* pv. *phaseolicola* ICMP2740 | 19543 | GCA_001400605.1 | 5.97 | LJQZ01 | 287 | 5107 | Scaffold | Thakur *et al.*, 2016 |
| *Pseudomonas syringae* pv. *syringae* B64 | 19543 | GCA_000331385.1 | 5.93 | ANZF01 | 1 | 4965 | Chromosome | Dudnik & Dudler, 2013a |
| *Pseudomonas syringae* pv. *syringae* SM | 19543 | GCA_000412165.1 | 6.12 | APWT01 | 2 | 5203 | Chromosome | Dudnik & Dudler, 2013b |
| *Pseudomonas syringae* pv. *maculicola* KN91 | 19543 | GCA_001294185.1 | 5.94 | LGLF01 | 55 | 4874 | Scaffold | Thakur *et al.*, 2016 |
| *Pseudomonas* *amygdali* pv. *eriobotryae* CFBP2343 | 19543 | GCA_001538055.1 | 6.32 | LIID01 | 128 | 5519 | Contig | Nowell *et al.*, 2016 |
| *Pseudomonas* *amygdali* pv. *aesculi* 2336 | 19543 | GCA_001538085.1 | 6.20 | LILL01 | 283 | 5305 | Contig | Nowell *et al.*, 2016 |
| *Pseudomonas syringae* pv. *maculicola* M4a | 19543 | GCA_001294305.1 | 6.44 | LGLE01 | 44 | 5813 | Scaffold | Thakur *et al.*, 2016 |
| *Pseudomonas syringae* pv. *actinidiae* ICMP9617 | 19543 | GCA_000658965.1 | 6.50 | AOKP01 | 2 | 5270 | Chromosome | McCann *et al.*, 2013 |
| *Pseudomonas* *savastanoi* pv. *retacarpa* CECT4861 | 19543 | GCA_002115545.1 | 5.73 | NBYW01 | 316 | 4920 | Scaffold | Dillon *et al.*, 2019 |
| *Pseudomonas* *amygdali* pv. *dendropanacis* CFBP3226 | 19543 | GCA_001538145.1 | 5.87 | LIIE01 | 218 | 4943 | Contig | Nowell *et al.*, 2016 |
| *Pseudomonas* *savastanoi* pv. *fraxini* 2315 | 19543 | GCA_002269785.1 | 5.89 | NIAW01 | 222 | 5054 | Scaffold | Moreno-Perez *et al.* 2019 (ds) |
| *Pseudomonas syringae* pv. *philadelphi* ICMP8903 | 19543 | GCA_001400595.1 | 6.15 | LJQY01 | 479 | 5307 | Scaffold | Thakur *et al.*, 2016 |
| *Pseudomonas* *savastanoi* Phi3 | 19543 | GCA_002269885.1 | 5.87 | NIAX01 | 218 | 5038 | Scaffold | Caballo-Ponce *et al.* 2019 (ds) |
| *Pseudomonas* *amygdali* pv. *morsprunorum* R19646 | 19543 | GCA_002905755.1 | 6.32 | MLEF01 | 191 | 5549 | Contig | Hulin *et al.* 2018 |
| *Pseudomonas* *savastanoi* pv. *nerii* ESC23 | 19543 | GCA_002270015.1 | 5.84 | NIAY01 | 229 | 5018 | Scaffold | Pintado *et al.* 2019 (ds) |
| *Pseudomonas syringae* pv. *avellanae* str. ISPaVe013 | 19543 | GCA_000302795.1 | 6.06 | AKCJ01 | 37 | 5184 | Scaffold | O’Brien *et al.*, 2012 |
| *Pseudomonas syringae* pv. *avellanae* str. ISPaVe037 | 19543 | GCA_000302815.1 | 5.88 | AKCK01 | 39 | 5102 | Scaffold | O’Brien *et al.*, 2012 |
| *Pseudomonas syringae* pv. *apii* ICMP2814 | 19543 | GCA_001401385.1 | 6.05 | LJPR01 | 203 | 5408 | Scaffold | Thakur *et al.*, 2016 |
| *Pseudomonas* *amygdali* pv. *lachrymans* 98A-744 | 19543 | GCA_001006445.1 | 6.28 | LCWT01 | 69 | 5524 | Scaffold | Jeong *et al.*, 2015 |
| *Pseudomonas syringae* BRIP39023 | 19543 | GCA_000333995.1 | 5.94 | AMZX01 | 34 | 5114 | Scaffold | Gardiner *et al.*, 2013 |
| *Pseudomonas syringae* pv. *berberidis* ICMP4116 | 19543 | GCA_001401405.1 | 6.26 | LJPU01 | 341 | 5366 | Scaffold | Thakur *et al.*, 2016 |
| *Pseudomonas* *amygdali* pv. *tabaci* str. ATCC11528 | 19543 | GCA_001006455.1 | 6.13 | LCWS01 | 18 | 5388 | Scaffold | Jeong *et al.*, 2015 |
| *Pseudomonas* *amygdali* pv. *lachrymans* 3988 | 19543 | GCA_001293835.1 | 6.06 | LGLJ01 | 128 | 5303 | Scaffold | Mott *et al.*, 2016 |
| *Pseudomonas syringae* BRIP34881 | 19543 | GCA_000334055.1 | 6.02 | AMXL01 | 96 | 5061 | Scaffold | Gardiner *et al.*, 2013 |
| *Pseudomonas syringae* pv. *syringae* Alf3 | 19543 | GCA_000738515.1 | 5.81 | JPNN01 | 29 | 4903 | Scaffold | Harrison *et al.*, 2016 |
| *Pseudomonas syringae* pv. *syringae* 642 | 19543 | GCA_000177515.1 | 5.81 | ADGB01 | 296 | 5055 | Contig | Clarke *et al.*, 2010 |
| *Pseudomonas* *amygdali* pv. *myricae* AZ84488 | 19543 | GCA_001293985.1 | 6.03 | LGLA01 | 62 | 5116 | Scaffold | Mott *et al.*, 2016 |
| *Pseudomonas* *amygdali* pv. *sesami* HC_1 | 19543 | GCA_001294115.1 | 6.17 | LGKS01 | 197 | 5283 | Scaffold | Mott *et al.*, 2016 |
| *Pseudomonas* *amygdali* pv. *hibisci* ICMP9623 | 19543 | GCA_001400395.1 | 6.11 | LJQN01 | 156 | 5334 | Scaffold | Thakur *et al.*, 2016 |
| *Pseudomonas* *amygdali* pv. *mellea* ICMP5711 | 19543 | GCA_001400875.1 | 5.90 | LJQS01 | 260 | 4927 | Scaffold | Thakur *et al.*, 2016 |
| *Pseudomonas* *amygdali* pv. *tabaci* ICMP5711 | 19543 | GCA_001401095.1 | 6.24 | LJRL01 | 170 | 5482 | Scaffold | Thakur *et al.*, 2016 |
| *Pseudomonas* *amygdali* pv. *ulmi* ICMP3962 | 19543 | GCA_001401165.1 | 6.24 | LJRQ01 | 455 | 5415 | Scaffold | Thakur *et al.*, 2016 |
| *Pseudomonas* *amygdali* pv. *sesami* ICMP763 | 19543 | GCA_001401265.1 | 6.03 | LJRG01 | 462 | 5244 | Scaffold | Thakur *et al.*, 2016 |
| *Pseudomonas* *amygdali* pv. *morsprunorum* NZIPFR-PS6 | 19543 | GCA_002736925.1 | 6.39 | LKCD01 | 267 | 5556 | Scaffold | Visnovsky *et al.*, 2016 |
| *Pseudomonas syringae* pv. *actinidiae* ICMP18807 | 19543 | GCA_000344535.1 | 6.13 | ANJL01 | 148 | 5345 | Contig | Butler *et al.*, 2013 |
| *Pseudomonas syringae* pv. *actinidiae* ICMP19098 | 19543 | GCA_000416545.1 | 6.31 | AOKE01 | 315 | 5499 | Contig | Sawada *et al.*, 2014 |
| *Pseudomonas* *amygdali* pv. *morsprunorum* R15300 | 19543 | GCA_002905875.2 | 6.65 | MLEN02 | 6 | 5554 | Scaffold | Hulin *et al.* 2018 |
| *Pseudomonas syringae* pv. *actinidiae* ICMP19072 | 19543 | GCA_000416885.1 | 6.01 | AOJW01 | 290 | 5316 | Contig | McCann *et al.*, 2013 |
| *Pseudomonas syringae* pv. *pisi* str. PP1 | 19543 | GCA_000452445.2 | 5.95 | AUZR02 | 256 | 5139 | Contig | Baltrus *et al.*, 2014a |
| *Pseudomonas syringae* pv. *syringae* 1212 | 19543 | GCA_000452465.2 | 6.16 | AVCR02 | 338 | 5276 | Contig | Baltrus *et al.*, 2014a |
| *Pseudomonas syringae* USA011 | 19543 | GCA_000452525.3 | 6.42 | AVDX02 | 197 | 5373 | Contig | Baltrus *et al.*, 2014b |
| *Pseudomonas syringae* CC457 | 19543 | GCA_000452585.2 | 5.84 | AVEB02 | 356 | 4963 | Contig | Baltrus *et al.*, 2014b |
| *Pseudomonas syringae* CC440 | 19543 | GCA_000452605.2 | 5.73 | AVEC02 | 354 | 4863 | Contig | Baltrus *et al.*, 2014b |
| *Pseudomonas syringae* CC1630 | 19543 | GCA_000452625.2 | 6.06 | AVED02 | 283 | 5394 | Contig | Baltrus *et al.*, 2014b |
| *Pseudomonas syringae* CC1629 | 19543 | GCA_000452645.2 | 5.93 | AVEE02 | 261 | 5246 | Contig | Baltrus *et al.*, 2014b |
| *Pseudomonas syringae* CC1524 | 19543 | GCA_000452745.2 | 5.83 | AVEK02 | 264 | 5111 | Contig | Baltrus *et al.*, 2014b |
| *Pseudomonas syringae* CC1513 | 19543 | GCA_000452765.2 | 5.73 | AVEL02 | 164 | 5081 | Contig | Baltrus *et al.*, 2014b |
| *Pseudomonas syringae* CC1466 | 19543 | GCA_000452785.2 | 5.59 | AVEM02 | 294 | 4819 | Contig | Baltrus *et al.*, 2014b |
| *Pseudomonas syringae* CC1458 | 19543 | GCA_000452805.2 | 5.80 | AVEN02 | 364 | 4889 | Contig | Baltrus *et al.*, 2014b |
| *Pseudomonas syringae* CC1417 | 19543 | GCA_000452825.2 | 5.65 | AVEO02 | 210 | 4967 | Contig | Baltrus *et al.*, 2014b |
| *Pseudomonas syringae* KCTC12500 | 19543 | GCA_000507185.2 | 6.15 | AYTM02 | 2 | 5137 | Contig | Jeong *et al.*, 2016 |
| *Pseudomonas syringae* UB0390 | 19543 | GCA_000737225.1 | 5.26 | JPQV01 | 80 | 4658 | Contig | Baltrus *et al.* 2014 (ds) |
| *Pseudomonas syringae* CEB003 | 19543 | GCA_000737235.1 | 6.68 | JPQT01 | 183 | 5960 | Contig | Baltrus *et al.* 2014 (ds) |
| *Pseudomonas syringae* GAW0119 | 19543 | GCA_000737245.1 | 5.82 | JPQU01 | 109 | 5054 | Contig | Baltrus *et al.* 2014 (ds) |
| *Pseudomonas syringae* pv. *syringae* 41a | 19543 | GCA_000935775.1 | 5.98 | JYHJ01 | 24 | 5094 | Contig | Bartoli *et al.*, 2015 |
| *Pseudomonas syringae* pv. *syringae* CRAFRU11 | 19543 | GCA_000972155.1 | 5.86 | ATSU01 | 179 | 4974 | Contig | Scortichini *et al.*, 2013 |
| *Pseudomonas syringae* pv. *syringae* CRAFRU12 | 19543 | GCA_000972195.1 | 5.93 | ATSV01 | 246 | 5060 | Contig | Scortichini *et al.*, 2013 |
| *Pseudomonas syringae* pv. *aceris* A10853 | 19543 | GCA_001270465.1 | 6.29 | LGAR01 | 196 | 5367 | Contig | Mott *et al.*, 2016 |
| *Pseudomonas syringae* ICMP11293 | 19543 | GCA_001466845.1 | 6.14 | LKEP01 | 60 | 5352 | Contig | Visnovsky *et al.*, 2016 |
| *Pseudomonas syringae* pv. *syringae* PD2766 | 19543 | GCA_001466965.1 | 5.47 | LKEM01 | 105 | 4743 | Contig | Visnovsky *et al.*, 2016 |
| *Pseudomonas syringae* ICMP19498 | 19543 | GCA_001467105.1 | 6.04 | LKCH01 | 161 | 5233 | Contig | Visnovsky *et al.*, 2016 |
| *Pseudomonas syringae* ICMP11292 | 19543 | GCA_001467365.1 | 5.81 | LKGU01 | 70 | 5147 | Contig | Visnovsky *et al.*, 2016 |
| *Pseudomonas syringae* pv. *actinidifoliorum*  CFBP8043 | 19543 | GCA_001497495.1 | 6.06 | LJFM01 | 176 | 5274 | Contig | Cunty *et al.*, 2016 |
| *Pseudomonas syringae* pv. *syringae* 2340 | 19543 | GCA_001535725.1 | 6.18 | LIHT01 | 96 | 5251 | Contig | Nowell *et al.*, 2016 |
| *Pseudomonas syringae* pv. *rhaphiolepidis* CFBP4220 | 19543 | GCA_001535835.1 | 5.62 | LIHV01 | 291 | 4725 | Contig | Nowell *et al.*, 2016 |
| *Pseudomonas syringae* pv. *syringae* 2339 | 19543 | GCA_001535855.1 | 6.12 | LIHU01 | 68 | 5174 | Contig | Nowell *et al.*, 2016 |
| *Pseudomonas syringae* pv. *papulans* CFBP1754 | 19543 | GCA_001535905.1 | 6.18 | LIHW01 | 173 | 5369 | Contig | Nowell *et al.*, 2016 |
| *Pseudomonas syringae* pv. *syringae* HRI-W 7924 | 19543 | GCA_001535945.1 | 6.24 | LIHR01 | 130 | 5305 | Contig | Nowell *et al.*, 2016 |
| *Pseudomonas syringae* pv. *broussonetiae* CFBP5140 | 19543 | GCA_001538205.1 | 6.11 | LIII01 | 357 | 5209 | Contig | Nowell *et al.*, 2016 |
| *Pseudomonas syringae* pv. *castaneae* CFBP4217 | 19543 | GCA_001538245.1 | 6.26 | LIIH01 | 218 | 5349 | Contig | Nowell *et al.*, 2016 |
| *Pseudomonas syringae* pv. *daphniphylli* CFBP4219 | 19543 | GCA_001538255.1 | 6.26 | LIIF01 | 355 | 5292 | Contig | Bartoli *et al.*, 2015 |
| *Pseudomonas syringae* pv. *syringae* MB03 | 19543 | GCA_001623415.1 | 5.78 | LAGV01 | 76 | 4935 | Contig | Ali *et al.*, 2016 |
| *Pseudomonas syringae* pv. *syringae* 2507 | 19543 | GCA_001675375.1 | 5.94 | LYUO01 | 97 | 4960 | Contig | Sultanov *et al.*, 2016 |
| *Pseudomonas syringae* pv. *syringae* 1845 | 19543 | GCA_001675415.1 | 5.77 | LYUP01 | 91 | 4795 | Contig | Sultanov *et al.*, 2016 |
| *Pseudomonas syringae* GR12-2 | 19543 | GCA_001698815.1 | 6.60 | LGSI01 | 74 | 5753 | Contig | Patten *et al.*, 2016 |
| *Pseudomonas syringae* pv. *actinidifoliorum* ICMP18802 | 19543 | GCA_002003545.1 | 6.42 | MUKM01 | 116 | 5612 | Contig | Butler *et al.* 2016 (ds) |
| *Pseudomonas syringae* pv. *actinidiae* C10 | 19543 | GCA_002174905.1 | 6.20 | MTHJ01 | 325 | 5356 | Contig | McCann *et al.*, 2017 |
| *Pseudomonas syringae* pv. *actinidiae* C17 | 19543 | GCA_002175005.1 | 6.34 | MTHQ01 | 366 | 5479 | Contig | McCann *et al.*, 2017 |
| *Pseudomonas* sp. CFII64 | 19543 | GCA_000416235.1 | 6.42 | ATLO01 | 54 | 5695 | Contig | Feris and lalor 2010 (ds) |
| *Pseudomonas syringae* RMX.24.a.1 | 19543 | GCA_002318665.1 | 6.27 | NHSS01 | 224 | 5230 | Contig | Karasov *et al.*, 2017 |
| *Pseudomonas syringae* NP29.1a | 19543 | GCA_002318725.1 | 6.09 | NHSX01 | 208 | 5185 | Contig | Karasov *et al.*, 2017 |
| *Pseudomonas syringae* Knox652c | 19543 | GCA_002318735.1 | 5.91 | NHSY01 | 160 | 4989 | Contig | Karasov *et al.*, 2017 |
| *Pseudomonas syringae* KN2.a.3 | 19543 | GCA_002318805.1 | 5.80 | NHTA01 | 133 | 4693 | Contig | Karasov *et al.*, 2017 |
| *Pseudomonas syringae* LMC.P91 | 19543 | GCA_002318815.1 | 6.32 | NHTB01 | 243 | 5354 | Contig | Karasov *et al.*, 2017 |
| *Pseudomonas syringae* LMC.P80 | 19543 | GCA_002318825.1 | 6.19 | NHTC01 | 137 | 5252 | Contig | Karasov *et al.*, 2017 |
| *Pseudomonas syringae* LMC.P10 | 19543 | GCA_002318875.1 | 6.24 | NHTD01 | 172 | 5289 | Contig | Karasov *et al.*, 2017 |
| *Pseudomonas syringae* LP868.1a | 19543 | GCA_002318895.1 | 5.91 | NHSU01 | 123 | 4996 | Contig | Karasov *et al.*, 2017 |
| *Pseudomonas syringae* NCPPB 3871 | 19543 | GCA_002699965.1 | 5.87 | LKEN01 | 78 | 5074 | Contig | Visnovsky *et al.*, 2016 |
| *Pseudomonas syringae* pv. *actinidiae* MAFF212054 | 19543 | GCA_002762615.1 | 6.54 | PESZ01 | 262 | 5645 | Contig | Poulter *et al.*, 2017 |
| *Pseudomonas syringae* pv. *syringae* Pss9644 | 19543 | GCA_002905835.1 | 6.17 | MLEK01 | 75 | 5202 | Contig | Hulin *et al.* 2018 |
| *Pseudomonas syringae* pv. *syringae* Pss9293 | 19543 | GCA_002905935.1 | 6.14 | MLEQ01 | 73 | 5288 | Contig | Hulin *et al.* 2018 |
| *Pseudomonas syringae* pv. *syringae* Pss9656 | 19543 | GCA_002905975.1 | 5.98 | MLEM01 | 39 | 5083 | Contig | Hulin *et al.* 2018 |
| *Pseudomonas syringae* pv. *syringae* Pss9654 | 19543 | GCA_002906015.1 | 5.94 | MLES01 | 49 | 5044 | Contig | Hulin *et al.* 2018 |
| *Pseudomonas syringae* BS0426 | 19543 | GCA_002906135.1 | 6.09 | PPYD01 | 41 | 5457 | Contig | Loper *et al.* 2018 (ds) |
| *Pseudomonas syringae* pv. *syringae* 5264 | 19543 | GCA_002916225.1 | 6.03 | NBAQ01 | 59 | 5216 | Contig | Hulin *et al.* 2018 |
| *Pseudomonas syringae* pv. *syringae* 7928A | 19543 | GCA_002916255.1 | 6.13 | NBAL01 | 59 | 5282 | Contig | Hulin *et al.* 2018 |
| *Pseudomonas syringae* pv. *syringae* 5275 | 19543 | GCA_002916275.1 | 5.99 | NBAP01 | 65 | 5120 | Contig | Hulin *et al.* 2018 |
| *Pseudomonas syringae* 7969 | 19543 | GCA_002916335.1 | 6.19 | NBAJ01 | 92 | 5299 | Contig | Hulin *et al.* 2018 |
| *Pseudomonas syringae* 7928C | 19543 | GCA_002916375.1 | 6.00 | NBAM01 | 51 | 5250 | Contig | Hulin *et al.* 2018 |
| *Pseudomonas syringae* pv. *syringae* 2676C | 19543 | GCA_002917155.1 | 6.16 | MLEY01 | 90 | 5260 | Contig | Hulin *et al.* 2018 |
| *Pseudomonas syringae* pv. *syringae* psy100 | 19543 | GCA_002917175.1 | 5.87 | MLEV01 | 23 | 5061 | Contig | Hulin *et al.* 2018 |
| *Pseudomonas syringae* pv. *syringae* 2675C | 19543 | GCA_002917195.1 | 5.99 | MLEX01 | 65 | 5076 | Contig | Hulin *et al.* 2018 |
| *Pseudomonas syringae* 03-A13 | 19543 | GCA_002939285.1 | 6.02 | MUJX01 | 122 | 5094 | Contig | Newberry *et al.* 2017 (ds) |
| *Pseudomonas syringae* pv. *pisi* 1456A | 19543 | GCA_003205935.1 | 6.46 | QJTW01 | 561 | 5441 | Contig | Payne *et al.* 2018 (ds) |
| *Pseudomonas syringae* pv. *pisi* 202 | 19543 | GCA_003205965.1 | 6.51 | QJTX01 | 417 | 5570 | Contig | Payne *et al.* 2018 (ds) |
| *Pseudomonas syringae* BS3827 | 19543 | GCA_900113625.1 | 6.08 | FOQB01 | 28 | 5240 | Contig | Varghese 2016 (ds) |
| *Pseudomonas syringae* BS2732 | 19543 | GCA_900114665.1 | 5.90 | FOTU01 | 69 | 5128 | Contig | Varghese 2016 (ds) |
| *Pseudomonas syringae* pv. *cerasicola* CFBP6110 | 19543 | GCA_900289135.1 | 5.91 | OLMP01 | 2 | 4948 | Contig | Ruinelli *et al.*, 2019 |
| *Pseudomonas syringae* pv. *theae* ICMP3923 | 19543 | GCA_000416465.2 | 6.25 | AOJV01 | 375 | 5471 | Scaffold | Thakur *et al.*, 2016 |
| *Pseudomonas syringae* pv. *actinidiae* ICMP19099 | 19543 | GCA_000416805.1 | 6.23 | AOKD01 | 342 | 5387 | Scaffold | McCann *et al.*, 2013 |
| *Pseudomonas* sp. ICMP10191 | 19543 | GCA_001467335.1 | 6.00 | LKGW01 | 189 | 5163 | Contig | Visnovsky *et al.*, 2016 |
| *Pseudomonas syringae* pv. *syringae* A2 | 19543 | GCA_001293665.1 | 5.90 | LGKU01 | 23 | 5040 | Scaffold | Mott *et al.*, 2016 |
| *Pseudomonas syringae* pv. *cilantro* 0788_9 | 19543 | GCA_001293775.1 | 5.92 | LGLN01 | 102 | 5054 | Scaffold | Mott *et al.*, 2016 |
| *Pseudomonas syringae* pv. *papulans* ICMP4048 | 19543 | GCA_001401005.1 | 6.10 | LJRB01 | 298 | 5223 | Scaffold | Thakur *et al.*, 2016 |
| *Pseudomonas syringae* pv. *aptata* ICMP459 | 19543 | GCA_001401335.1 | 5.98 | LJRP01 | 149 | 5088 | Scaffold | Thakur *et al.*, 2016 |
| *Pseudomonas syringae* pv. *syringae* PD2774 | 19543 | GCA_001466875.1 | 6.36 | LKEL01 | 180 | 5377 | Scaffold | Visnovsky *et al.*, 2016 |
| *Pseudomonas syringae* ICMP13102 | 19543 | GCA_001466945.1 | 5.95 | LKEO01 | 158 | 5025 | Scaffold | Visnovsky *et al.*, 2016 |
| *Pseudomonas syringae* ICMP19499 | 19543 | GCA_001467115.1 | 6.16 | LKCI01 | 102 | 5357 | Scaffold | Visnovsky *et al.*, 2016 |
| *Pseudomonas syringae* pv. *actinidiae* ICMP19497 | 19543 | GCA_001467225.1 | 6.32 | LKBQ01 | 233 | 5537 | Scaffold | Visnovsky *et al.*, 2016 |
| *Pseudomonas syringae* ICMP11168 | 19543 | GCA_001467285.1 | 5.97 | LKGV01 | 97 | 5080 | Scaffold | Visnovsky *et al.*, 2016 |
| *Pseudomonas syringae* pv. *actinidifoliorum* CFBP8039 | 19543 | GCA_001497595.1 | 6.10 | LJJM01 | 204 | 5308 | Scaffold | Cunty *et al.*, 2016 |
| *Pseudomonas syringae* pv. *actinidiae* K4 | 19543 | GCA_002111835.1 | 6.06 | MTYT01 | 258 | 5388 | Scaffold | McCann *et al.*, 2017 |
| *Pseudomonas syringae* pv. *actinidiae* C3 | 19543 | GCA_002174875.1 | 6.24 | MTCQ01 | 318 | 5492 | Scaffold | McCann *et al.*, 2017 |
| *Pseudomonas syringae* pv. *actinidiae* C16 | 19543 | GCA_002174995.1 | 6.39 | MTHP01 | 386 | 5545 | Scaffold | McCann *et al.*, 2017 |
| *Pseudomonas syringae* ICMP3690 | 19543 | GCA_002699825.1 | 6.34 | LKBV01 | 311 | 5493 | Scaffold | Visnovsky *et al.*, 2016 |
| *Pseudomonas syringae* ICMP4122 | 19543 | GCA_002699845.1 | 5.97 | LKBX01 | 39 | 5141 | Scaffold | Visnovsky *et al.*, 2016 |
| *Pseudomonas syringae* ICMP8406 | 19543 | GCA_002699885.1 | 6.30 | LKBZ01 | 425 | 5404 | Scaffold | Visnovsky *et al.*, 2016 |
| *Pseudomonas syringae* pv. *cerasicola* ICMP13929 | 19543 | GCA_002700065.1 | 5.67 | LKCB01 | 385 | 4712 | Scaffold | Visnovsky *et al.*, 2016 |
| *Pseudomonas syringae* pv. *actinidiae* M401 | 19543 | GCA_002890475.1 | 6.26 | MDXG01 | 292 | 5474 | Scaffold | Zhao *et al.*, 2019 |
| *Pseudomonas syringae* pv. *actinidiae* M256 | 19543 | GCA_002890485.1 | 6.22 | MDXH01 | 266 | 5495 | Scaffold | Zhao *et al.*, 2019 |
| *Pseudomonas syringae* BS2900 | 19543 | GCA_900107345.1 | 6.21 | FNPP01 | 47 | 5558 | Scaffold | Varghese 2016 (ds) |
| *Pseudomonas syringae* BS3829 | 19543 | GCA_900113535.1 | 5.97 | FOPR01 | 38 | 5129 | Scaffold | Varghese 2016 (ds) |
| *Pseudomonas syringae* BS0292 | 19543 | GCA_900115275.1 | 6.23 | FOVV01 | 75 | 5338 | Scaffold | Varghese 2016 (ds) |
| *Pseudomonas syringae* pv. *panici* str. LMG2367 | 19543 | GCA_000282735.1 | 5.99 | ALAC01 | 148 | - | Contig | Liu *et al.*, 2012 |
| *Pseudomonas* sp. BS3767 | 19543 | GCA_900099665.1 | 6.14 | FNCX01 | 22 | 5265 | Contig | Varghese 2016 (ds) |
| *Pseudomonas* sp. ICMP3272 | 19543 | GCA_001466905.1 | 5.93 | LKEK01 | 129 | 5135 | Scaffold | Visnovsky *et al.*, 2016 |
| *Pseudomonas* sp. ICMP561 | 19543 | GCA_002699985.1 | 6.31 | LKBT01 | 70 | 5566 | Scaffold | Visnovsky *et al.*, 2016 |
| *Pseudomonas* sp. NP28-5 | 19543 | GCA_003201385.1 | 5.90 | QICP01 | 56 | 5025 | Scaffold | Pelletier 2018 (ds) |
| *Pseudomonas* sp. NP10-3 | 19543 | GCA_003253455.1 | 6.05 | QKUC01 | 52 | 5136 | Scaffold | Pelletier 2018 (ds) |
| *Pseudomonas* sp. NFACC10-1 | 19543 | GCA_900119195.1 | 5.83 | FPJB01 | 21 | 4957 | Scaffold | Varghese 2016 (ds) |
| *Pseudomonas* sp. Irchel 3A18 | 19543 | GCA_900187465.1 | 5.92 | FYDI01 | 37 | 5012 | Scaffold | Butaite *et al.*, 2017 |
| *Pseudomonas* sp. Irchel 3A5 | 19543 | GCA_900187575.1 | 6.43 | FYDU01 | 34 | 5694 | Scaffold | Butaite *et al.*, 2017 |
| *Pseudomonas syringae* Riq4 | 19546 | GCA_001238485.1 | 6.36 | LFQK01 | 131 | 5627 | Contig | Chan *et al.* 2015 (ds) |

(ds) direct submission to genbank

Ali M, Sun Y, Xie L, Yu H, Bashir A, Li L, 2016. The pathogenicity of *Pseudomonas syringae* MB03 against *Caenorhabditis elegans* and the transcriptional response of nematicidal genes upon different nutritional conditions. *Frontiers in Microbiology* **7**, 1–12.

Baltrus DA, Dougherty K, Beckstrom-Sternberg SM, Beckstrom-Sternberg JS, Foster JT, 2014a. Incongruence between multi-locus sequence analysis (MLSA) and whole-genome-based phylogenies: *Pseudomonas syringae* pathovar *pisi* as a cautionary tale. *Molecular plant pathology* **15**, 461–465.

Baltrus DA, Nishimura MT, Romanchuk A et al., 2011. Dynamic evolution of pathogenicity revealed by sequencing and comparative genomics of 19 *Pseudomonas syringae* isolates. *PLoS Pathogens* **7**, 22.

Baltrus DA, Yourstone S, Lind A et al., 2014b. Draft genome sequences of a phylogenetically diverse suite of *Pseudomonas syringae* strains from multiple source populations. *Genome Announcements* **2**, e01195-13-e01195-13.

Bartoli C, Carrere S, Lamichhane R, Varvaro L, Morris CE, 2015. Whole-genome sequencing of 10 *Pseudomonas syringae* strains representing different host range spectra. *Genome Announcements* **3**, 2–3.

Besaury L, Amato P, Wirgot N, Sancelme M, Delort AM, 2017. Draft genome sequence of *Pseudomonas graminis* PDD-13b-3, a model strain isolated from cloud water. *Genome Announcements* **5**, 1–2.

Buell CR, Joardar V, Lindeberg M et al., 2003. The complete genome sequence of the *Arabidopsis* and tomato pathogen *Pseudomonas syringae* pv. *tomato* DC3000. *PNAS* **100**, 10181–10186.

Busquets A, Gomila M, Beiki F et al., 2017. *Pseudomonas caspiana* sp. nov., a citrus pathogen in the *Pseudomonas syringae* phylogenetic group. *Systematic and applied microbiology* **40**, 266—273.

Butaite E, Baumgartner M, Wyder S, Kümmerli R, 2017. Siderophore cheating and cheating resistance shape competition for iron in soil and freshwater *Pseudomonas* communities. *Nature Communications* **8**.

Butler MI, Stockwell P a, Black M a, Day RC, Lamont IL, Poulter RTM, 2013. *Pseudomonas syringae* pv. actinidiae from recent outbreaks of kiwifruit bacterial canker belong to different clones that originated in China*. PloS one* **8,** e57464.

Clarke CR, Cai R, Studholme DJ, Guttman DS, Vinatzer B a, 2010. *Pseudomonas syringae* strains naturally lacking the classical *P. syringae* hrp/hrc Locus are common leaf colonizers equipped with an atypical type III secretion system. *Molecular plant-microbe interactions MPMI* **23**, 198–210.

Cunty A, Cesbron S, Briand M et al., 2016. Draft genome sequences of five *Pseudomonas syringae* pv. *actinidifoliorum* strains isolated in France. *Brazilian Journal of Microbiology* **47**, 529–530.

Dillon MM, Thakur S, Almeida RND, Wang PW, Weir BS, Guttman DS, 2019. Recombination of ecologically and evolutionarily significant loci maintains genetic cohesion in the *Pseudomonas syringae* species complex *Genome Biology* **20**, 1–28.

Dudnik A, Dudler R, 2013a. Non contiguous-finished genome sequence of *Pseudomonas syringae* pathovar *syringae* strain B64 isolated from wheat. *Standards in Genomic Sciences* **8**, 420–429.

Dudnik A, Dudler R, 2013b. High-Quality Draft Genome Sequence of *Pseudomonas syringae* pv . *syringae* Strain SM , Isolated from Wheat. *Genome Announcements* **1**, 6–7.

Feil H, Feil WS, Chain P et al., 2005. Comparison of the complete genome sequences of *Pseudomonas syringae* pv. *syringae* B728a and pv. *tomato* DC3000. *PNAS* **102**, 11064–11069.

Firrao G, Torelli E, Polano C et al., 2018. Genomic structural variations affecting virulence during clonal expansion of *Pseudomonas syringae* pv. *actinidiae* biovar 3 in Europe. *Frontiers in Microbiology* **9**.

Fujikawa T, Sawada H, 2016. Genome analysis of the kiwifruit canker pathogen *Pseudomonas syringae* pv. *actinidiae* biovar 5. *Scientific Reports* **6**, 1–11.

Gardiner DM, Stiller J, Covarelli L, Lindeberg M, Shivas RG, Manners JM, 2013. Genome sequences of *Pseudomonas* spp. isolated from cereal crops. *Genome announcements* **1**, e00209–e00213.

Green S, Studholme DJ, Laue BE et al., 2010. Comparative genome analysis provides insights into the evolution and adaptation of *Pseudomonas syringae* pv. *aesculi* on *Aesculus hippocastanum*. *PloS one* **5**, e10224.

Haney CH, Wiesmann CL, Shapiro LR et al., 2018. Rhizosphere-associated *Pseudomonas* induce systemic resistance to herbivores at the cost of susceptibility to bacterial pathogens. *Molecular Ecology* **27**, 1833–1847.

Harrison J, Dornbusch MR, Samac D, Studholme DJ, 2016. Draft genome sequence of *Pseudomonas syringae* pv. *syringae* ALF3 isolated from alfalfa. *Genome Announcements* **4**, 2015–2016.

Hockett KL, Nishimura MT, Karlsrud E, Dougherty K, Baltrus DA, 2014. *Pseudomonas syringae* CC1557: A Highly Virulent Strain With an Unusually Small Type III Effector Repertoire That Includes a Novel Effector. *Molecular plant-microbe interactions MPMI* **27**, 923–32.

Jeong H, Kloepper J, Ryu C-M, 2015. Genome Sequences of *Pseudomonas amygdali* pv. *tabaci* Strain ATCC 11528 and pv. *lachrymans* Strain 98A-744. *Genome Announcements* **1**, 2–3.

Jeong H, Lee D-H, Ryu C-M, Park S-H, 2016. Toward complete bacterial genome sequencing through the combined use of multiple Next-generation Sequencing Platforms. *Journal of microbiology and biotechnology* **26**, 207–212.

Joardar V, Lindeberg M, Jackson RW et al., 2005. Whole-genome sequence analysis of *Pseudomonas syringae* pv. *phaseolicola* 1448A reveals divergence among pathovars in genes involved in virulence and transposition. *Journal of Bacteriology* **187**, 6488–6498.

Jones LA, Saha S, Collmer A, Smart CD, Lindeberg M, 2015. Genome-assisted development of a diagnostic protocol for distinguishing high virulence *Pseudomonas syringae* pv. *tomato* strains. *Plant Disease* **99,** 527–534.

Kałużna M, Willems A, Pothier JF, Ruinelli M, Sobiczewski P, Puławska J, 2016. *Pseudomonas cerasi* sp. nov. (non Griffin, 1911) isolated from diseased tissue of cherry. *Systematic and Applied Microbiology* **39,** 370–377.

Karasov TL, Barrett L, Hershberg R, Bergelson J, 2017. Similar levels of gene content variation observed for *Pseudomonas syringae* populations extracted from single and multiple host species. *PLoS ONE* **12**, 1–18.

Karasov T, Horton M, Bergelson J, 2014. Genomic variability as a driver of plant–pathogen coevolution? *Current opinion in plant biology* **18**, 24–30.

Kong J, Jiang H, Li B, Zhao W, Li Z, Zhu S, 2016. Complete genome sequence of *Pseudomonas syringae* pv. *lapsa* strain ATCC 10859, isolated from infected wheat. *Genome Announcements* **4**, 4–5.

Li L, Yuan L, Shi Y et al., 2019. Comparative genomic analysis of *Pseudomonas amygdali* pv. *lachrymans* NM002: Insights into its potential virulence genes and putative invasion determinants. *Genomics* **111**, 1493–1503.

Liu H, Qiu H, Zhao W et al., 2012. Genome sequence of the plant pathogen *Pseudomonas syringae* pv. *panici* LMG 2367. *Journal of Bacteriology* **194**, 5693–5694.

Marcelletti S, Scortichini M, 2014. Definition of Plant-Pathogenic Pseudomonas Genomospecies of the *Pseudomonas syringae* Complex Through Multiple Comparative Approaches. *Phytopathology* **104**, 1274–1282.

Martínez-García PM, Rodríguez-Palenzuela P, Arrebola E et al., 2015. Bioinformatics analysis of the complete genome sequence of the mango tree pathogen *Pseudomonas syringae* pv. *syringae* UMAF0158 reveals traits relevant to virulence and epiphytic lifestyle. *PLoS ONE* **10**, 1–26.

McCann HC, Li L, Liu Y et al., 2017. Origin and evolution of the kiwifruit canker pandemic. *Genome Biology and Evolution* **9**, 932–944.

McCann HC, Rikkerink EH a, Bertels F et al., 2013. Genomic Analysis of the Kiwifruit Pathogen *Pseudomonas syringae* pv. *actinidiae* Provides Insight into the Origins of an Emergent Plant Disease. *PLoS pathogens* **9**, e1003503.

Moretti C, Cortese C, Passos da Silva D et al., 2014. Draft genome sequence of *Pseudomonas* *savastanoi* pv. *savastanoi* strain DAPP-PG 722, isolated in italy from an olive plant affected by knot disease. *Genome Announcements* **2**, e00864-14-e00864-14.

Mott GA, Thakur S, Smakowska E et al., 2016. Genomic screens identify a new phytobacterial microbe-associated molecular pattern and the cognate *Arabidopsis* receptor-like kinase that mediates its immune elicitation. *Genome Biology* **17,** 98.

Nowell RW, Laue BE, Sharp PM, Green S, 2016. Comparative genomics reveals genes significantly associated with woody hosts in the plant pathogen *Pseudomonas syringae*. *Molecular Plant Pathology* **17**, 1409–1424.

O’Brien HE, Thakur S, Gong Y et al., 2012. Extensive remodeling of the *Pseudomonas syringae* pv. avellanae type III secretome associated with two independent host shifts onto hazelnut. *BMC microbiology* **12**, 141.

Patten CL, Jeong H, Blakney AJC, Wallace N, 2016. Draft genome sequence of a diazotrophic, plant growth-promoting rhizobacterium of the *Pseudomonas syringae* complex. *Genome Announcements* **4**, 41–42.

Poulter R, Taiaroa G, Sumpter N, Stockwell P, Butler M, 2017. Complete genome sequence of the kiwifruit pathogen *Pseudomonas syringae* pv. *actinidiae* biovar 5, originating from Japan. *Genome Announcements* **5**, 17–18.

Qi M, Wang D, Bradley CA, Zhao Y, 2011. Genome sequence analyses of *Pseudomonas savastanoi* pv. *glycinea* and subtractive hybridization-based comparative genomics with nine pseudomonads. *PLoS ONE* **6**, e16451.

Ramkumar G, Lee SW, Weon HY, Kim BY, Lee YH, 2015. First report on the whole genome sequence of *Pseudomonas cichorii* strain JBC1 and comparison with other *Pseudomonas* species. *Plant Pathology* **64**, 63–70.

Ravindran A, Jalan N, Yuan JS, Wang N, Gross DC, 2015. Comparative genomics of *Pseudomonas syringae* pv. *syringae* strains B301D and HS191 and insights into intrapathovar traits associated with plant pathogenesis. *MicrobiologyOpen* **4,** 553–573.

Rodríguez-Palenzuela P, Matas IM, Murillo J et al., 2010. Annotation and overview of the *Pseudomonas savastanoi* pv. *savastanoi* NCPPB 3335 draft genome reveals the virulence gene complement of a tumour-inducing pathogen of woody hosts. *Environmental microbiology* **12**, 1604–1620.

Rombouts S, Van Vaerenbergh J, Volckaert A et al., 2016. Isolation and characterization of *Pseudomonas syringae* pv. *porri* from leek in Flanders. *European Journal of Plant Pathology* **144**, 185–198.

Ruinelli M, Blom J, Pothier J, 2017. Complete Genome Sequence of Pseudomonas viridiflava CFBP 1590, Isolated from Diseased Cherry in France. *Genome Announcements* **5**, 1–2.

Ruinelli M, Blom J, Smits THM, Pothier JF, 2019. Comparative genomics and pathogenicity potential of members of the *Pseudomonas syringae* species complex on *Prunus* spp. *BMC Genomics* **20**, 1–16.

Samad A, Trognitz F, Antonielli L, Compant S, Sessitsch A, 2016. High-quality draft genome sequence of an endophytic Pseudomonas viridiflava strain with herbicidal properties against its host, the weed *Lepidium draba* L. *Genome Announcements* **4**, 4–5.

Sawada H, Miyoshi T, Ide T, 2014. Novel MLSA group (Psa5) of *Pseudomonas syringae* pv. *actinidiae* causing bacterial canker of kiwifruit (*Actinidia chinensis*) in Japan. Japanese Journal of *Phytopathology* **80**, 171–184.

Scortichini M, Marcelletti S, Ferrante P, Firrao G, 2013. A Genomic Redefinition of *Pseudomonas* *avellanae* species. *PLoS ONE* **8**, 1–16.

Sultanov RI, Arapidi GP, Vinogradova S V., Govorun VM, Luster DG, Ignatov AN, 2016. Comprehensive analysis of draft genomes of two closely related *Pseudomonas syringae* phylogroup 2b strains infecting mono- and dicotyledon host plants. *BMC Genomics* **17**.

Thakur S, Weir BS, Guttman D, 2016. Phytopathogen genome announcement: Draft genome sequences of 62 *Pseudomonas syringae* type and pathotype strains. *Molecular Plant-Microbe Interactions MPMI* **29**, 243–246.

Timilsina S, Minsavage G V., Preston J et al., 2018. *Pseudomonas floridensis* sp. nov., a bacterial pathogen isolated from tomato. *International Journal of Systematic and Evolutionary Microbiology* **68**, 64–70.

Visnovsky SB, Fiers M, Lu A, Panda P, Taylor R, Pitman AR, 2016. Draft genome sequences of 18 strains of *Pseudomonas* isolated from kiwifruit plants in New Zealand and overseas. *Genome Announcements* **4**, e00061–e00016.

Zhao Z, Chen J, Gao X et al., 2019. Comparative genomics reveal pathogenicity-related loci in *Pseudomonas syringae* pv. *actinidiae* biovar 3. *Molecular Plant Pathology* **20**, 923–942.
