# Supplementary material for: Cherry picking by pseudomonads: After a century of research on canker, genomics provides insights into the evolution of pathogenicity towards stone fruits
Source: Plant Pathol. 2020 May 6;69(6):962–78. doi: 10.1111/ppa.13189 (PMC7386918; doi:10.1111/ppa.13189)
Supplement: Supplementary file 2 — Table S2 [file PPA-69-962-s002.docx]

Table S2: Known Type 3 Effectors present in representative complete genomes of *Prunus* pathogens with genomic location

| Strain | Effector | Location | Length | Start | End | Strand | Notes |
| --- | --- | --- | --- | --- | --- | --- | --- |
| R1-5244 | *avrD1* | p2 | 311 | 103281 | 104213 | - |  |
| R1-5244 | *avrE1* | Chromosome | 1715 | 1692847 | 1697991 | - |  |
| R1-5244 | *avrRpm2* | p2 | 223 | 22823 | 23491 | + |  |
| R1-5244 | *hopA1* | p1 | 375 | 56049 | 57173 | - |  |
| R1-5244 | *hopA2* | Chromosome | 364 | 1724323 | 1725414 | + | premature stop codon |
| R1-5244 | *hopAA1* | Chromosome | 339 | 1686552 | 1687568 | + | partial hit |
| R1-5244 | *hopAC1* | Chromosome | 1812 | 746785 | 752220 | - | partial hit |
| R1-5244 | *hopAE1* | Chromosome | 912 | 5165123 | 5167858 | - |  |
| R1-5244 | *hopAF1* | Chromosome | 284 | 4622846 | 4623697 | + |  |
| R1-5244 | *hopAF1* | p2 | 280 | 90481 | 91320 | + |  |
| R1-5244 | *hopAH1* | Chromosome | 416 | 3742036 | 3743283 | + |  |
| R1-5244 | *hopAJ1* | Chromosome | 374 | 1006026 | 1007147 | - | partial hit |
| R1-5244 | *hopAJ1* | Chromosome | 445 | 5243797 | 5245131 | + |  |
| R1-5244 | *hopAK1* | Chromosome | 524 | 4660324 | 4661895 | + |  |
| R1-5244 | *hopAN1* | Chromosome | 429 | 661777 | 663063 | - |  |
| R1-5244 | *hopAO2* | Chromosome | 186 | 115180 | 115737 | - | partial hit |
| R1-5244 | *hopAO2* | Chromosome | 186 | 209639 | 210196 | - | partial hit |
| R1-5244 | *hopAR1* | Chromosome | 267 | 3418055 | 3418855 | + |  |
| R1-5244 | *hopAS1* | Chromosome | 1361 | 5579294 | 5583376 | + |  |
| R1-5244 | *hopAT1* | Chromosome | 82 | 1008113 | 1008358 | - |  |
| R1-5244 | *hopAU1* | Chromosome | 731 | 4270964 | 4273156 | - |  |
| R1-5244 | *hopAV1* | Chromosome | 806 | 218262 | 220679 | - |  |
| R1-5244 | *hopAY1* | Chromosome | 324 | 177351 | 178322 | + |  |
| R1-5244 | *hopAY1* | Chromosome | 204 | 4277113 | 4277724 | **-** | partial hit |
| R1-5244 | *hopAZ1* | Chromosome | 194 | 2244062 | 2244643 | - |  |
| R1-5244 | *hopBD1* | p2 | 300 | 46724 | 47623 | + |  |
| R1-5244 | *hopBF1* | p2 | 192 | 94534 | 95109 | + |  |
| R1-5244 | *hopBK1* | Chromosome | 55 | 3416268 | 3416432 | - | partial hit |
| R1-5244 | *hopBL2* | Chromosome | 752 | 251498 | 253753 | + |  |
| R1-5244 | *hopBL2* | Chromosome | 752 | 5394014 | 5396269 | - |  |
| R1-5244 | *hopD1* | Chromosome | 705 | 356358 | 358472 | + |  |
| R1-5244 | *hopF3* | Chromosome | 196 | 166454 | 167041 | - |  |
| R1-5244 | *hopF3* | Chromosome | 198 | 4288812 | 4289405 | - |  |
| R1-5244 | *hopF4* | Chromosome | 205 | 256406 | 257020 | - |  |
| R1-5244 | *hopI1* | Chromosome | 336 | 5209296 | 5210303 | - |  |
| R1-5244 | *hopJ1* | Chromosome | 149 | 1467706 | 1468152 | - |  |
| R1-5244 | *hopM1* | Chromosome | 543 | 1690704 | 1692332 | + | partial hit |
| R1-5244 | *hopQ1* | Chromosome | 447 | 358594 | 359934 | - |  |
| R1-5244 | *hopR1* | Chromosome | 1959 | 348114 | 353990 | - |  |
| R1-5244 | *hopV1* | Chromosome | 393 | 3477476 | 3478654 | + |  |
| R1-5244 | *hopZ4* | Chromosome | 335 | 1080971 | 1081975 | - |  |
| R1-5300 | *avrA1* | Chromosome | 906 | 4560032 | 4562749 | - |  |
| R1-5300 | *avrD1* | p3 | 311 | 63009 | 63941 | - |  |
| R1-5300 | *avrE1* | Chromosome | 1712 | 4235573 | 4240708 | - |  |
| R1-5300 | *avrPto1* | Chromosome | 162 | 2360936 | 2361421 | - |  |
| R1-5300 | *avrRpm2* | Chromosome | 223 | 2784919 | 2785587 | + |  |
| R1-5300 | *hopA2* | Chromosome | 364 | 4267042 | 4268133 | + | premature stop codon |
| R1-5300 | *hopAA1* | Chromosome | 486 | 4229222 | 4230679 | + |  |
| R1-5300 | *hopAB1* | Chromosome | 541 | 2913198 | 2914820 | - |  |
| R1-5300 | *hopAC1* | Chromosome | 1812 | 3337886 | 3343321 | - | partial hit |
| R1-5300 | *hopAE1* | Chromosome | 912 | 1648699 | 1651434 | - |  |
| R1-5300 | *hopAF1* | Chromosome | 284 | 1066915 | 1067766 | + |  |
| R1-5300 | *hopAH1* | Chromosome | 416 | 182437 | 183684 | + |  |
| R1-5300 | *hopAJ1* | Chromosome | 445 | 1728430 | 1729764 | + |  |
| R1-5300 | *hopAK1* | Chromosome | 524 | 1102780 | 1104351 | + |  |
| R1-5300 | *hopAN1* | Chromosome | 429 | 3253056 | 3254342 | - |  |
| R1-5300 | *hopAO1* | p2 | 220 | 44370 | 45029 | + | partial hit |
| R1-5300 | *hopAO2* | Chromosome | 186 | 2761967 | 2762524 | - | partial hit |
| R1-5300 | *hopAR1* | Chromosome | 267 | 654193 | 654993 | + |  |
| R1-5300 | *hopAS1* | Chromosome | 1361 | 2064434 | 2068516 | + |  |
| R1-5300 | *hopAT1* | Chromosome | 65 | 1882615 | 1882809 | - | partial hit |
| R1-5300 | *hopAU1* | Chromosome | 727 | 714432 | 716612 | - |  |
| R1-5300 | *hopAY1* | Chromosome | 207 | 720560 | 721180 | - | partial hit |
| R1-5300 | *hopAZ1* | Chromosome | 194 | 4896649 | 4897230 | - |  |
| R1-5300 | *hopBB1* | p1 | 283 | 29543 | 30391 | - |  |
| R1-5300 | *hopBK1* | Chromosome | 55 | 652406 | 652570 | - | partial hit |
| R1-5300 | *hopBL2* | Chromosome | 522 | 1879361 | 1880926 | - | partial hit |
| R1-5300 | *hopD1* | Chromosome | 705 | 2907854 | 2909968 | + |  |
| R1-5300 | *hopD1* | Chromosome | 705 | 4532272 | 4534386 | + |  |
| R1-5300 | *hopF3* | Chromosome | 198 | 731358 | 731951 | - |  |
| R1-5300 | *hopG1* | p1 | 443 | 46480 | 47808 | + |  |
| R1-5300 | *hopI1* | Chromosome | 336 | 1694069 | 1695076 | - |  |
| R1-5300 | *hopJ1* | Chromosome | 149 | 4009282 | 4009728 | - |  |
| R1-5300 | *hopM1* | Chromosome | 636 | 4233139 | 4235046 | + | partial hit |
| R1-5300 | *hopQ1* | Chromosome | 447 | 2910090 | 2911430 | - |  |
| R1-5300 | *hopQ1* | Chromosome | 226 | 4536860 | 4537537 | - | partial hit |
| R1-5300 | *hopR1* | Chromosome | 1959 | 2899610 | 2905486 | - |  |
| R1-5300 | *hopV1* | Chromosome | 393 | 5632070 | 5633248 | - |  |
| R1-5300 | *hopW1* | Chromosome | 776 | 524716 | 527043 | - |  |
| R1-5300 | *hopX1* | p1 | 384 | 22465 | 23616 | - |  |
| R1-5300 | *hopX1* | Chromosome | 384 | 2748774 | 2749925 | + | premature stop codon |
| R1-3840 | *avrE1* | Chromosome | 1712 | 4444303 | 4449438 | + |  |
| R1-3840 | *hopA1* | p2 | 375 | 18381 | 19505 | - |  |
| R1-3840 | *hopA2* | Chromosome | 364 | 4416878 | 4417969 | - | premature stop codon |
| R1-3840 | *hopAA1* | Chromosome | 344 | 4454753 | 4455784 | - | partial hit |
| R1-3840 | *hopAC1* | Chromosome | 1812 | 5309292 | 5314727 | + | partial hit |
| R1-3840 | *hopAE1* | Chromosome | 912 | 932868 | 935603 | + |  |
| R1-3840 | *hopAF1* | Chromosome | 282 | 1476713 | 1477558 | - |  |
| R1-3840 | *hopAF1* | p4 | 195 | 77763 | 78347 | + | partial hit |
| R1-3840 | *hopAH1* | Chromosome | 415 | 2356517 | 2357761 | - |  |
| R1-3840 | *hopAJ1* | Chromosome | 445 | 855594 | 856928 | - |  |
| R1-3840 | *hopAJ1* | Chromosome | 374 | 5054369 | 5055490 | + | partial hit |
| R1-3840 | *hopAK1* | Chromosome | 524 | 1436461 | 1438032 | - |  |
| R1-3840 | *hopAN1* | Chromosome | 429 | 5398450 | 5399736 | + |  |
| R1-3840 | *hopAO2* | Chromosome | 186 | 5880558 | 5881115 | + |  |
| R1-3840 | *hopAR1* | Chromosome | 267 | 2684185 | 2684985 | - |  |
| R1-3840 | *hopAS1* | Chromosome | 1361 | 518564 | 522646 | - |  |
| R1-3840 | *hopAT1* | Chromosome | 82 | 5053158 | 5053403 | + |  |
| R1-3840 | *hopAU1* | Chromosome | 731 | 1826630 | 1828822 | + |  |
| R1-3840 | *hopAV1* | Chromosome | 806 | 5842319 | 5844736 | + |  |
| R1-3840 | *hopAY1* | Chromosome | 323 | 5986124 | 5987092 | - |  |
| R1-3840 | *hopAY1* | Chromosome | 207 | 1822062 | 1822682 | + | partial hit |
| R1-3840 | *hopAY1* | p3 | 323 | 34570 | 35538 | + |  |
| R1-3840 | *hopAZ1* | Chromosome | 229 | 3897537 | 3898223 | + |  |
| R1-3840 | *hopBB1* | p1 | 283 | 29484 | 30332 | - |  |
| R1-3840 | *hopBD1* | p4 | 300 | 41349 | 42248 | - |  |
| R1-3840 | *hopBF1* | p4 | 192 | 73974 | 74549 | - |  |
| R1-3840 | *hopBK1* | Chromosome | 55 | 2686608 | 2686772 | + |  |
| R1-3840 | *hopBL1* | p1 | 721 | 84626 | 86788 | + |  |
| R1-3840 | *hopBL2* | Chromosome | 752 | 704458 | 706713 | + |  |
| R1-3840 | *hopBL2* | Chromosome | 752 | 5807198 | 5809453 | - |  |
| R1-3840 | *hopD1* | Chromosome | 705 | 5703047 | 5705161 | - |  |
| R1-3840 | *hopF3* | Chromosome | 198 | 1809515 | 1810108 | + |  |
| R1-3840 | *hopF3* | p2 | 194 | 8559 | 9140 | + |  |
| R1-3840 | *HopF4* | Chromosome | 205 | 5803931 | 5804545 | + |  |
| R1-3840 | *hopI1* | Chromosome | 336 | 890423 | 891430 | + |  |
| R1-3840 | *hopJ1* | Chromosome | 149 | 4675242 | 4675688 | + |  |
| R1-3840 | *hopM1* | Chromosome | 542 | 4449965 | 4451590 | - |  |
| R1-3840 | *hopQ1* | Chromosome | 447 | 5701585 | 5702925 | + |  |
| R1-3840 | *hopR1* | Chromosome | 1920 | 5707529 | 5713288 | + |  |
| R1-3840 | *hopV1* | Chromosome | 393 | 2622335 | 2623513 | - |  |
| R2leaf | *avrB2* | p5 | 352 | 16270 | 17325 | + |  |
| R2leaf | *avrE1* | Chromosome | 1801 | 1540943 | 1546345 | - |  |
| R2leaf | *avrRps4* | Chromosome | 221 | 3065037 | 3065699 | - |  |
| R2leaf | *hopA2* | Chromosome | 377 | 2199556 | 2200686 | - |  |
| R2leaf | *hopAA1* | Chromosome | 343 | 1534614 | 1535642 | + | partial hit |
| R2leaf | *hopAB3* | Chromosome | 440 | 2775221 | 2776540 | + | partial hit |
| R2leaf | *hopAC1* | Chromosome | 2022 | 5468761 | 5474826 | + | partial hit |
| R2leaf | *hopAF1* | p1 | 280 | 76234 | 77073 | + |  |
| R2leaf | *hopAF1* | p3 | 280 | 23832 | 24671 | - |  |
| R2leaf | *hopAG1* | Chromosome | 487 | 1042345 | 1043805 | + | partial hit |
| R2leaf | *hopAH1* | Chromosome | 420 | 1043909 | 1045168 | + |  |
| R2leaf | *hopAH1* | Chromosome | 415 | 2528654 | 2529898 | - |  |
| R2leaf | *hopAI1* | Chromosome | 267 | 1045335 | 1046135 | + |  |
| R2leaf | *hopAJ1* | Chromosome | 414 | 962978 | 964219 | - |  |
| R2leaf | *hopAJ1* | Chromosome | 445 | 5261578 | 5262912 | + |  |
| R2leaf | *hopAK1* | Chromosome | 555 | 4639961 | 4641625 | + |  |
| R2leaf | *hopAN1* | Chromosome | 429 | 5574001 | 5575287 | + |  |
| R2leaf | *hopAO1* | p2 | 305 | 20247 | 21161 | + | partial hit |
| R2leaf | *hopAR1* | Chromosome | 267 | 5928972 | 5929772 | + |  |
| R2leaf | *hopAS1* | Chromosome | 1358 | 527993 | 532066 | - |  |
| R2leaf | *hopAT1* | Chromosome | 82 | 965062 | 965307 | - |  |
| R2leaf | *hopAU1* | p1 | 731 | 85723 | 87915 | + |  |
| R2leaf | *hopAY1* | p1 | 323 | 94635 | 95603 | - |  |
| R2leaf | *hopAY1* | p2 | 323 | 42596 | 43564 | + |  |
| R2leaf | *hopAZ1* | p2 | 217 | 25139 | 25789 | - |  |
| R2leaf | *hopBB1* | p4 | 283 | 40368 | 41216 | - |  |
| R2leaf | *hopBD1* | p4 | 300 | 15840 | 16739 | + |  |
| R2leaf | *hopBF1* | p3 | 192 | 5174 | 5749 | - |  |
| R2leaf | *hopBN1* | Chromosome | 293 | 4002883 | 4003761 | - | partial hit |
| R2leaf | *hopD1* | p1 | 705 | 62424 | 64538 | + | premature stop codon |
| R2leaf | *hopE1* | Chromosome | 211 | 1819950 | 1820582 | + |  |
| R2leaf | *hopF2* | Chromosome | 215 | 1576937 | 1577581 | - |  |
| R2leaf | *HopF4* | Chromosome | 205 | 989482 | 990096 | + |  |
| R2leaf | *hopH1* | Chromosome | 218 | 984173 | 984826 | - |  |
| R2leaf | *hopI1* | Chromosome | 487 | 5229069 | 5230529 | - |  |
| R2leaf | *hopJ1* | Chromosome | 149 | 1324015 | 1324461 | - |  |
| R2leaf | *hopL1* | Chromosome | 909 | 3001130 | 3003856 | + |  |
| R2leaf | *hopM1* | Chromosome | 712 | 1538227 | 1540362 | + |  |
| R2leaf | *hopN1* | Chromosome | 350 | 1532209 | 1533258 | + |  |
| R2leaf | *hopP1* | Chromosome | 324 | 3295742 | 3296713 | - |  |
| R2leaf | *hopR1* | Chromosome | 1957 | 1006811 | 1012681 | + |  |
| R2leaf | *hopS2* | Chromosome | 177 | 5180865 | 5181395 | - |  |
| R2leaf | *hopW1* | Chromosome | 767 | 1002414 | 1004714 | + |  |
| R2leaf | *hopX1* | p5 | 343 | 2 | 1030 | - |  |
| R2leaf | *hopY1* | Chromosome | 287 | 149577 | 150437 | - |  |
| R2-3800 | *avrE1* | Chromosome | 1801 | 1702564 | 1707966 | - |  |
| R2-3800 | *avrRps4* | Chromosome | 204 | 558936 | 559547 | - | partial hit |
| R2-3800 | *hopA2* | Chromosome | 377 | 2346170 | 2347300 | - |  |
| R2-3800 | *hopAA1* | Chromosome | 343 | 1696235 | 1697263 | + | partial hit |
| R2-3800 | *hopAB3* | Chromosome | 440 | 270021 | 271340 | + | partial hit |
| R2-3800 | *hopAC1* | Chromosome | 2022 | 2073248 | 2079313 | + |  |
| R2-3800 | *hopAF1* | p1 | 280 | 13598 | 14437 | + |  |
| R2-3800 | *hopAG1* | Chromosome | 487 | 1206005 | 1207465 | + | partial hit |
| R2-3800 | *hopAH1* | Chromosome | 415 | 21730 | 22974 | - |  |
| R2-3800 | *hopAH1* | Chromosome | 255 | 1208061 | 1208825 | + | partial hit |
| R2-3800 | *hopAI1* | Chromosome | 261 | 1209013 | 1209795 | + |  |
| R2-3800 | *hopAJ1* | Chromosome | 374 | 1094714 | 1095835 | - | partial hit |
| R2-3800 | *hopAJ1* | Chromosome | 445 | 1863577 | 1864911 | + |  |
| R2-3800 | *hopAK1* | Chromosome | 555 | 1249815 | 1251479 | + |  |
| R2-3800 | *hopAN1* | Chromosome | 429 | 2177034 | 2178320 | + |  |
| R2-3800 | *hopAO1* | p2 | 305 | 52837 | 53751 | + |  |
| R2-3800 | *hopAR1* | Chromosome | 267 | 2530975 | 2531775 | + |  |
| R2-3800 | *hopAS1* | Chromosome | 1358 | 624656 | 628729 | - |  |
| R2-3800 | *hopAT1* | Chromosome | 82 | 1096801 | 1097046 | - |  |
| R2-3800 | *hopAU1* | Chromosome | 731 | 137078 | 139270 | - |  |
| R2-3800 | *hopAY1* | Chromosome | 323 | 129390 | 130358 | + |  |
| R2-3800 | *hopAZ1* | p2 | 194 | 57729 | 58310 | - |  |
| R2-3800 | *hopBB1* | p1 | 280 | 58716 | 59555 | + |  |
| R2-3800 | *hopBD1* | p1 | 300 | 39951 | 40850 | - |  |
| R2-3800 | *hopBN1* | Chromosome | 293 | 626263 | 627141 | - | partial hit |
| R2-3800 | *hopD1* | Chromosome | 705 | 59958 | 62072 | - | premature stop codon |
| R2-3800 | *hopE1* | Chromosome | 211 | 1978431 | 1979063 | + |  |
| R2-3800 | *hopF2* | Chromosome | 215 | 1738558 | 1739202 | - |  |
| R2-3800 | *HopF4* | Chromosome | 205 | 1149743 | 1150357 | + |  |
| R2-3800 | *hopH1* | Chromosome | 218 | 1133159 | 1133812 | - |  |
| R2-3800 | *hopI1* | Chromosome | 148 | 1831884 | 1832327 | - | partial hit |
| R2-3800 | *hopJ1* | Chromosome | 149 | 1486816 | 1487262 | - |  |
| R2-3800 | *hopL1* | Chromosome | 909 | 495015 | 497741 | + |  |
| R2-3800 | *hopM1* | Chromosome | 712 | 1699848 | 1701983 | + |  |
| R2-3800 | *hopN1* | Chromosome | 350 | 1693830 | 1694879 | + |  |
| R2-3800 | *hopO1* | p2 | 283 | 21335 | 22183 | + |  |
| R2-3800 | *hopP1* | Chromosome | 324 | 787702 | 788673 | - |  |
| R2-3800 | *hopR1* | Chromosome | 1957 | 1170471 | 1176341 | + |  |
| R2-3800 | *hopS2* | Chromosome | 177 | 1782807 | 1783337 | - |  |
| R2-3800 | *hopT1* | p2 | 379 | 22192 | 23328 | + |  |
| R2-3800 | *hopW1* | Chromosome | 768 | 1166074 | 1168377 | + |  |
| R2-3800 | *hopY1* | Chromosome | 287 | 243317 | 244177 | - |  |
| Pss-9097 | *avrE1* | Chromosome | 1820 | 1345632 | 1351091 | - |  |
| Pss-9097 | *avrRpm1* | Chromosome | 228 | 853911 | 854594 | - |  |
| Pss-9097 | *hopAC1* | Chromosome | 2023 | 597281 | 603349 | - |  |
| Pss-9097 | *hopAE1* | Chromosome | 912 | 4965411 | 4968146 | - |  |
| Pss-9097 | *hopAN1* | Chromosome | 429 | 511347 | 512633 | - |  |
| Pss-9097 | *hopAP1* | Chromosome | 230 | 2037950 | 2038639 | - |  |
| Pss-9097 | *hopAW1* | Chromosome | 220 | 2044725 | 2045384 | - | premature stop codon |
| Pss-9097 | *hopH1* | Chromosome | 218 | 2036313 | 2036966 | - |  |
| Pss-9097 | *hopI1* | Chromosome | 336 | 5056609 | 5057616 | - |  |
| Pss-9097 | *hopJ1* | Chromosome | 150 | 1147193 | 1147642 | - |  |
| Pss-9097 | *hopAI1* | Chromosome | 247 | 898580 | 899320 | + |  |
| Pss-9097 | *hopA2* | Chromosome | 382 | 1380832 | 1381977 | + |  |
| Pss-9097 | *hopAA1* | Chromosome | 553 | 1339100 | 1340758 | + |  |
| Pss-9097 | *hopAF1* | Chromosome | 284 | 4390982 | 4391833 | + |  |
| Pss-9097 | *hopAG1* | Chromosome | 716 | 894817 | 896964 | + |  |
| Pss-9097 | *hopAH1* | Chromosome | 427 | 897073 | 898353 | + |  |
| Pss-9097 | *hopAH1* | Chromosome | 380 | 3606299 | 3607438 | + |  |
| Pss-9097 | *hopAJ1* | Chromosome | 445 | 5088131 | 5089465 | + |  |
| Pss-9097 | *hopAK1* | Chromosome | 543 | 4450436 | 4452064 | + |  |
| Pss-9097 | *hopAR1* | Chromosome | 267 | 5684243 | 5685043 | + |  |
| Pss-9097 | *hopBE1* | Chromosome | 588 | 4478743 | 4480506 | + |  |
| Pss-9097 | *hopM1* | Chromosome | 718 | 1342923 | 1345076 | + |  |
| cera-58T | *avrE1* | Chromosome | 1789 | 1271183 | 1276549 | - |  |
| cera-58T | *avrRps4* | p1 | 221 | 20995 | 21657 | + |  |
| cera-58T | *avrRps4* | p3 | 221 | 41199 | 41861 | - |  |
| cera-58T | *hopAA1* | Chromosome | 483 | 1264934 | 1266382 | + |  |
| cera-58T | *hopAA1* | p3 | 487 | 58570 | 60030 | - |  |
| cera-58T | *hopAB1* | p5 | 257 | 48346 | 49116 | + | partial hit |
| cera-58T | *hopAC1* | Chromosome | 2022 | 5248622 | 5254687 | + |  |
| cera-58T | *hopAE1* | Chromosome | 912 | 4771892 | 4774627 | - |  |
| cera-58T | *hopAF1* | Chromosome | 280 | 4819625 | 4820464 | - |  |
| cera-58T | *hopAG1* | Chromosome | 715 | 777636 | 779780 | + |  |
| cera-58T | *hopAH1* | Chromosome | 427 | 779892 | 781172 | + |  |
| cera-58T | *hopAH1* | Chromosome | 415 | 2377418 | 2378662 | - |  |
| cera-58T | *hopAI1* | Chromosome | 156 | 781342 | 781809 | + | partial hit |
| cera-58T | *hopAJ1* | Chromosome | 445 | 4863549 | 4864883 | + |  |
| cera-58T | *hopAK1* | Chromosome | 524 | 4276534 | 4278105 | + |  |
| cera-58T | *hopAN1* | Chromosome | 429 | 5339312 | 5340598 | + |  |
| cera-58T | *hopAO2* | Chromosome | 338 | 719217 | 720230 | - |  |
| cera-58T | *hopAO2* | p1 | 224 | 74603 | 75274 | - | partial hit |
| cera-58T | *hopAR1* | Chromosome | 267 | 230479 | 231279 | - |  |
| cera-58T | *hopAU1* | p2 | 731 | 24474 | 26666 | + |  |
| cera-58T | *hopAV1* | p2 | 806 | 13158 | 15575 | + |  |
| cera-58T | *hopAW1* | Chromosome | 219 | 4815266 | 4815922 | - | partial hit |
| cera-58T | *hopAY1* | Chromosome | 323 | 4820648 | 4821616 | - |  |
| cera-58T | *hopAZ1* | Chromosome | 194 | 3887057 | 3887638 | + |  |
| cera-58T | *hopBB1* | p5 | 283 | 958 | 1806 | + |  |
| cera-58T | *hopBF1* | p4 | 192 | 11765 | 12340 | - |  |
| cera-58T | *hopBI1* | p5 | 397 | 37142 | 38332 | + | partial hit |
| cera-58T | *hopBM1* | p5 | 157 | 59578 | 60048 | - |  |
| cera-58T | *hopBN1* | Chromosome | 293 | 3773369 | 3774247 | - | partial hit |
| cera-58T | *hopD1* | p2 | 711 | 6224 | 8356 | + |  |
| cera-58T | *hopG1* | Chromosome | 515 | 710257 | 711801 | - |  |
| cera-58T | *hopI1* | Chromosome | 337 | 4830030 | 4831040 | - |  |
| cera-58T | *hopJ1* | Chromosome | 150 | 1084046 | 1084495 | - |  |
| cera-58T | *hopM1* | Chromosome | 530 | 1269038 | 1270627 | + | partial hit |
| cera-58T | *hopO1* | p1 | 283 | 85940 | 86788 | + |  |
| cera-58T | *hopR1* | p2 | 1735 | 35281 | 40485 | + | partial hit |
| cera-58T | *hopT1* | p1 | 379 | 86797 | 87933 | + |  |
| cera-58T | *hopW1* | Chromosome | 774 | 1532203 | 1534509 | + |  |
| cera-58T | *hopX1* | Chromosome | 384 | 4826041 | 4827192 | + |  |
| avii-3846 | *avrB4* | p5 | 320 | 28815 | 29774 | - |  |
| avii-3846 | *avrD1* | p5 | 311 | 689 | 1621 | - |  |
| avii-3846 | *avrE1* | Chromosome | 1801 | 1489543 | 1494945 | - |  |
| avii-3846 | *avrRps4* | p3 | 221 | 57233 | 57895 | + |  |
| avii-3846 | *avrRps4* | p4 | 221 | 9951 | 10613 | + |  |
| avii-3846 | *hopA1* | Chromosome | 380 | 300009 | 301148 | - |  |
| avii-3846 | *hopAA1* | Chromosome | 323 | 1480473 | 1481441 | + | partial hit |
| avii-3846 | *hopAA1* | p3 | 487 | 43836 | 45296 | + |  |
| avii-3846 | *hopAB1* | p4 | 257 | 27264 | 28034 | - | partial hit |
| avii-3846 | *hopAB3* | Chromosome | 394 | 2580719 | 2581900 | + | partial hit |
| avii-3846 | *hopAC1* | Chromosome | 1140 | 707168 | 710587 | - | partial hit |
| avii-3846 | *hopAF1* | p2 | 213 | 23236 | 23874 | - |  |
| avii-3846 | *hopAG1* | Chromosome | 399 | 5137921 | 5139117 | - | partial hit |
| avii-3846 | *hopAH1* | Chromosome | 415 | 2352603 | 2353847 | - |  |
| avii-3846 | *hopAH1* | Chromosome | 422 | 5135642 | 5136907 | - |  |
| avii-3846 | *hopAI1* | Chromosome | 267 | 5134672 | 5135472 | - |  |
| avii-3846 | *hopAJ1* | Chromosome | 445 | 922397 | 923731 | - |  |
| avii-3846 | *hopAK1* | Chromosome | 550 | 4490694 | 4492343 | + |  |
| avii-3846 | *hopAN1* | Chromosome | 429 | 616186 | 617472 | - |  |
| avii-3846 | *hopAO1* | p3 | 268 | 108038 | 108841 | + | partial hit |
| avii-3846 | *hopAR1* | Chromosome | 268 | 5173015 | 5173818 | + |  |
| avii-3846 | *hopAS1* | Chromosome | 1364 | 5742995 | 5747086 | + |  |
| avii-3846 | *hopAU1* | Chromosome | 731 | 282809 | 285001 | + |  |
| avii-3846 | *hopAY1* | Chromosome | 323 | 259893 | 260861 | - |  |
| avii-3846 | *hopAY1* | p3 | 323 | 21465 | 22433 | + |  |
| avii-3846 | *hopAZ1* | Chromosome | 227 | 3883694 | 3884374 | + |  |
| avii-3846 | *hopAZ1* | p3 | 217 | 4088 | 4738 | - |  |
| avii-3846 | *hopB1* | Chromosome | 466 | 1521446 | 1522843 | + |  |
| avii-3846 | *hopBB1* | Chromosome | 283 | 5195314 | 5196162 | + |  |
| avii-3846 | *hopBD1* | p2 | 300 | 36909 | 37808 | - |  |
| avii-3846 | *hopBF1* | p2 | 192 | 27277 | 27852 | + |  |
| avii-3846 | *hopBN1* | Chromosome | 298 | 3300089 | 3300982 | - |  |
| avii-3846 | *hopD1* | Chromosome | 705 | 5588185 | 5590299 | + |  |
| avii-3846 | *hopI1* | Chromosome | 280 | 962780 | 963619 | + | partial hit |
| avii-3846 | *hopJ1* | Chromosome | 148 | 4812728 | 4813171 | + |  |
| avii-3846 | *hopL1* | Chromosome | 909 | 2881911 | 2884637 | - |  |
| avii-3846 | *hopM1* | Chromosome | 712 | 1483608 | 1485743 | + | premature stop codon |
| avii-3846 | *hopN1* | Chromosome | 350 | 1476124 | 1477173 | + |  |
| avii-3846 | *hopO1* | Chromosome | 230 | 1018554 | 1019243 | + | partial hit |
| avii-3846 | *hopO1* | Chromosome | 301 | 1020768 | 1021670 | + |  |
| avii-3846 | *hopO1* | p1 | 283 | 18884 | 19732 | - |  |
| avii-3846 | *hopP1* | Chromosome | 324 | 3096119 | 3097090 | - |  |
| avii-3846 | *hopQ1* | Chromosome | 447 | 5590421 | 5591761 | - |  |
| avii-3846 | *hopR1* | Chromosome | 1957 | 5160261 | 5166131 | - |  |
| avii-3846 | *hopS1* | Chromosome | 122 | 1016221 | 1016586 | + |  |
| avii-3846 | *hopS2* | Chromosome | 177 | 1022735 | 1023265 | + |  |
| avii-3846 | *hopT1* | Chromosome | 389 | 1019252 | 1020418 | + |  |
| avii-3846 | *hopT1* | p1 | 379 | 17739 | 18875 | - |  |
| avii-3846 | *hopX1* | Chromosome | 384 | 5205865 | 5207016 | + |  |
| avii-3846 | *hopY1* | Chromosome | 287 | 148626 | 149486 | - |  |
