# Supplementary material for: Cherry picking by pseudomonads: After a century of research on canker, genomics provides insights into the evolution of pathogenicity towards stone fruits
Source: Plant Pathol. 2020 May 6;69(6):962–78. doi: 10.1111/ppa.13189 (PMC7386918; doi:10.1111/ppa.13189)
Supplement: Supplementary file 3 — Table S3 [file PPA-69-962-s003.docx]

Table S3: Protein IDs of sequences used for tBLASTn analysis of the canonical Type 3 Secretion System in *Pseudomonas syringae*

| **Protein ID** | **Description** | **Organism** |
| --- | --- | --- |
| AAO54900 | membrane-bound lytic murein transglycosylase D | *Pseudomonas syringae* pv. *tomato* DC3000 |
| AAO54901 | type III transcriptional regulator HrpR | *Pseudomonas syringae* pv. *tomato* DC3000 |
| AAO54902 | type III transcriptional regulator HrpS | *Pseudomonas syringae* pv. *tomato* DC3000 |
| AAO54903 | type III helper protein HrpA1 | *Pseudomonas syringae* pv. *tomato* DC3000 |
| AAO54904 | type III helper protein HrpZ1 | *Pseudomonas syringae* pv. *tomato* DC3000 |
| AAO54905 | type III secretion protein HrpB | *Pseudomonas syringae* pv. *tomato* DC3000 |
| AAO54906 | type III secretion protein HrcJ | *Pseudomonas syringae* pv. *tomato* DC3000 |
| AAO54907 | type III secretion protein HrpD | *Pseudomonas syringae* pv. *tomato* DC3000 |
| AAO54908 | type III secretion protein HrpE | *Pseudomonas syringae* pv. *tomato* DC3000 |
| AAO54909 | type III secretion protein HrpF | *Pseudomonas syringae* pv. *tomato* DC3000 |
| AAO54910 | type III secretion protein HrpG | *Pseudomonas syringae* pv. *tomato* DC3000 |
| AAO54911 | outer-membrane type III secretion protein HrcC | *Pseudomonas syringae* pv. *tomato* DC3000 |
| AAO54912 | type III secretion protein HrpT | *Pseudomonas syringae* pv. *tomato* DC3000 |
| AAO54913 | negative regulator of hrp expression HrpV | *Pseudomonas syringae* pv. *tomato* DC3000 |
| AAO54926 | RNA polymerase sigma factor HrpL | *Pseudomonas syringae* pv. *tomato* DC3000 |
| AAO54927 | type III helper protein HrpK1 | *Pseudomonas syringae* pv. *tomato* DC3000 |
