# Supplementary material for: Cherry picking by pseudomonads: After a century of research on canker, genomics provides insights into the evolution of pathogenicity towards stone fruits
Source: Plant Pathol. 2020 May 6;69(6):962–78. doi: 10.1111/ppa.13189 (PMC7386918; doi:10.1111/ppa.13189)
Supplement: Supplementary file 4 — Text S1 [file PPA-69-962-s004.docx]

**Supplementary methods**

The maximum-likelihood phylogenetic tree in Fig. 3 was constructed using IQTREE (Nguyen *et al.*, 2015) from a 189,348bp protein alignment consisting of 899 concatenated single-copy core genes from 234 genomes. The model used was JTT+I+G with 1000 bootstraps. *Pseudomonas* sp. Riq4 (GCA_001238485.1) was used as an out-group. The tree was visualized and annotated using R packages ape and ggtree (Paradis *et al.*, 2004; Yu *et al.*, 2017). Clades coloured on the phylogeny indicate clustering by average nucleotide identity (ANI) with PYANI (Pritchard *et al.*, 2016) to show those strains with ≥95% identity. Scale shows substitutions per site.

Identification of a putatively complete T3SS for each strain was performing using tBLASTn of proteins in Table S3 representing the canonical T3SS in model strain *P. syringae* pv. *tomato* DC3000. If ≥80% of genes were present with ≥50% query length covered and ≥50% amino acid identity, the strain was concluded to likely possess the canonical T3SS. Toxin biosynthesis clusters were identified using a similar approach with all known genes involved needing to be present with ≥50% query length covered and ≥50% amino acid identity. T3 effector identification was as in Hulin *et al.* (2018). The total number of known full length effector alleles in each strain (not counting putative pseudogenes, multiple copies of effectors or those that span a contig break) was used to create the heatmap in Fig. 3.

The program circos (Krzywinski, 2009) was utilised to visualise the *P. syringae* pv. *morsprunorum* R1-5244 complete genome in Fig. 4. Analysis was as in Hulin *et al.* (2018). Type 3 Effectors (T3Es) were identified using tBLASTn of previously characterised T3Es from pseudomonas-syringae.org. The same was done for toxin biosynthesis genes. Genomic islands were identified using IslandViewer4 (Bertelli *et al.*, 2017) and prophage regions were identified using PHASTER (Arndt *et al.*, 2016). Locations of genes/regions were extracted from output files using bash scripting and used as input to circos. Effector horizontal gene transfer analysis was performed as in Hulin *et al.* (2018) by visualising effector protein phylogenies. Those in which the effector sequence clustered divergent cherry clades together show evidence of past transfers. DNA alignments presented in Fig. 4 were performed on Geneious V7 software (Kearse *et al.*, 2012) using MAFFT (Katoh *et al.*, 2002).

The venn diagram in Fig. 4 was created using an online tool available at <http://bioinformatics.psb.ugent.be/webtools/Venn/>

Arndt D, Grant JR, Marcu A *et al.*, 2016. PHASTER: A better, faster version of the PHAST phage search tool. *Nucleic acids research* **44**, W16–W21.

Bertelli C, Laird MR, Williams KP *et al.*, 2017. IslandViewer 4: Expanded prediction of genomic islands for larger-scale datasets. *Nucleic Acids Research* **45**, W30–W35.

Hulin MT, Armitage AD, Vicente JG *et al.*, 2018. Comparative genomics of Pseudomonas syringae reveals convergent gene gain and loss associated with specialization onto cherry (Prunus avium). *New Phytologist* **219**, 672–696.

Katoh K, Misawa K, Kuma K, Miyata T, 2002. MAFFT: A novel method for rapid multiple sequence alignment based on fast Fourier transform. *Nucleic acids research* **30**, 3059–3066.

Kearse M, Moir R, Wilson A *et al.*, 2012. Geneious Basic: An integrated and extendable desktop software platform for the organization and analysis of sequence data. *Bioinformatics* **28**, 1647–1649.

Krzywinski M et al, 2009. Circos: an Information Aesthetic for Comparative Genomics. *Genome Res* **19**, 1639–1645.

Nguyen LT, Schmidt HA, Von Haeseler A, Minh BQ, 2015. IQ-TREE: A fast and effective stochastic algorithm for estimating maximum-likelihood phylogenies. *Molecular Biology and Evolution* **32**, 268–274.

Paradis E, Claude J, Strimmer K, 2004. APE: Analyses of phylogenetics and evolution in R language. *Bioinformatics* **20**, 289–290.

Pritchard L, Glover RH, Humphris S, Elphinstone JG, Toth IK, 2016. Genomics and taxonomy in diagnostics for food security: Soft-rotting enterobacterial plant pathogens. *Analytical Methods* **8**, 12–24.

Yu G, Smith D, Zhu H, Guan Y, Lam T, 2017. ggtree: An R package for visualization and annotation of phylogenetic trees with their covariates and other associated data. *Methods in Ecology and Evolution* **8**, 28–36.
